# A study on food-medicine continuum among the non-institutionally trained *siddha* practitioners of Tiruvallur district, Tamil Nadu, India

**DOI:** 10.1186/s13002-018-0240-9

**Published:** 2018-06-28

**Authors:** S. Esakkimuthu, S. Sylvester Darvin, S. Mutheeswaran, M. Gabriel Paulraj, P. Pandikumar, S. Ignacimuthu, N. A. Al-Dhabi

**Affiliations:** 1 Division of Ethnopharmacology, Entomology Research Institute, Loyola College, Chennai, Tamil Nadu 600034 India; 2 International Scientific Partnership Programme, King Saud University, Riyadh, 11451 Saudi Arabia; 3 Addiriyah Chair for Environmental Studies, College of Science, King Saud University, 2455, Riyadh, 11451 Saudi Arabia

**Keywords:** Medicinal foods, Functional foods, Traditional brand identity, Indian traditional medicine

## Abstract

**Background:**

Medicinal properties of the food species are one of the poorly documented and important areas of ethnopharmacology. The present survey quantitatively documented the medicinal foods prescribed by the non-institutionally trained *siddha* practitioners of Tiruvallur district of Tamil Nadu.

**Methods:**

Field work was carried out between December 2014 and April 2017 using a questionnaire. The illnesses mentioned by the informants were grouped as illness categories on the basis of *emic* perceptions. Sufficiency of sampling of this survey was assessed by plotting the cumulative number of UR and Shannon-Wiener’s index. The indices such as informant consensus factor (FIC), Index of Agreement on Remedies (IAR), and Cultural Food Significance Index (CFSI) were calculated.

**Results:**

This study documented 165 medicinal foods used by 82 non-institutionally trained *siddha* practitioners of Tiruvallur district, and 73.93% of these preparations were plant based. Among the animal taxa, 82.05% were represented by fish taxa. The illness category gastrointestinal ailments is the majorly cited illness category treated with plant-based formulations. The illness categories viz., gastrointestinal ailments, hemorrhoids, and neural ailments had high consensus under the group of plant-based medicinal foods. In animal-based medicinal foods, *kapha* ailments had gained 23.07% of UR. The illness categories such as bone fractures, male reproductive ailments, blood ailments, and anabolic had high FIC values.

**Conclusions:**

Deeper studies on different dietary cultures of India may help to derive better interpretations on food-medicine continuum. This study identified some important claims such as the use of citron, pomegranate and *Solanum americanum* (gastrointestinal ailments), *Abutilon indicum*, onions and elephant foot yam (hemorrhoids), *Boerhavia diffusa* (urinary ailments), *Moringa oleifera* (anemia), *Aloe vera* (gynecological ailments), *Eclipta prostrata* (liver ailments), ivy gourd (diabetes), citron (hypertension), *Centella asiatica* (psychological ailments), spade nose shark (lactogogue), reticulate whipray (wheezing and bronchitis), *Katelysia opima* (impotence), Indian squid (anemia), and Indian oil sardine (anabolic). More studies on these claims will help identify novel functional foods to add to the field of medical nutrition therapy, with traditional brand identity. Robust studies on the documentation of the traditional knowledge on marine resources will yield a good database for various stakeholders and policy makers.

**Electronic supplementary material:**

The online version of this article (10.1186/s13002-018-0240-9) contains supplementary material, which is available to authorized users.

## Background

In many traditions, species which are used as medicine are also used as food and vice-versa; in many cases, ethnobiologists have documented this unclear delineation between food and medicine [1]. Ethnopharmacological surveys showed that the local people who gathered various wild species for food had knowledge about the health benefits of those species [2]. These health benefits go beyond from nutritional perspectives to health promoting phytochemicals [3] and many of them have been consumed by healthy people as a prophylactic measure. Local gastronomies are one of the important markers of regional identity and have been built upon various ecological, cultural, and religious beliefs [4]. Studies on the dietary patterns of a culture yield better clues on patterns of health and diseases observed in that population [5]. The importance of food-medicine interface in ethnopharmacology has been recognized for nearly 25 years [6, 7]. Many studies focused either on the dietary or pharmacological properties of the species and only limited studies addressed this food-medicine continuum [8].

India has a wide diversity of traditional cuisines; they contain whole grains, millets, wild gathered vegetables [9] and various spices for coloring, flavoring and preserving foods [10]. Besides cultural and religious beliefs, Indian cuisine was largely influenced by the principles of traditional medicinal systems. According to Indian philosophy, food was considered as the gift from god and it was classified into various categories [11]. The cuisine of *Tamils* is one of the important and oldest cuisine systems of India, and it is influenced largely by *siddha* system of traditional medicine [12]. The *sangam* (300BC–300AD) and post-*sangam* age (300–600AD) literatures such as *tirukkuṟaḷ*, *pattuppāṭṭu*, and *ācārakkōvai* describe various types foods and rules for eating. A classical *siddha* literature, *patārtta kuṇa cintāmaṇi*, describes the medicinal properties of various foods and drinks [13].

Diet diversification is one of the concepts to combat micronutrient malnutrition and to prevent chronic diseases [14, 15]. Various factors such as increasing healthcare costs, life expectancy, and desire for improving the quality of life among elders created a demand in global food industry to find novel foods with functional properties [16]. Globally, a significant increase in the consumption of functional foods for prevention and management of various chronic ailments was recorded. The market size of functional foods is expected to grow by 241 billion US$ by 2019. In India, the functional food industry is expected to grow by 20% and to reach a market size of 6.1 billion rupees by 2019–2020. Traditional diets are generally considered as holistic, healthy, and medicinal; thus, the demand and public interest on such foods are increasing rapidly, as in the case of herbal drugs [17]. Developing functional foods from traditional claims has been considered as an area for identifying novel functional foods [18]. In countries like India where traditional medicinal systems are looked with a nationalistic sentiment, the demand and the scope for functional foods with traditional brand identity are increasing. Besides these advantages, previous studies also demonstrated the adverse interactions of some functional foods with conventional therapies [19, 20]. In such scenario, it becomes important to document and inclusively evaluate the nutritional as well as functional properties of medicinal foods used locally and also to know the illnesses generally treated with these foods [8].

*Siddha* is one of the major traditional medical systems of India, which shares some commonalities with *ayurveda*. It has been majorly practiced in Tamil Nadu state and in its fringes; it has also been practiced in foreign countries such as Sri Lanka and Malaysia by Tamil people [21]. Previous studies indicated that the *siddha* literatures used in contemporary practice started from fourth to fifth century AD, though the practices emerged earlier [22]. The name *siddha* had also been coined recently in 1923 to delineate it from other systems of traditional medicines [23]. Literatures related to *siddha* are found exclusively in Tamil language as palm leaf manuscripts, and many of them remain undocumented. Institutional training on *siddha* system of medicine has been provided by the Government of India; however, the number of non-institutionally trained practitioners remains high [24]. Non-institutional training in *siddha* system of medicine is usually given from father to son and master to disciple forms [23]. This kind of knowledge transmission is usually done orally, and a lot of their recipes remain undocumented. Our previous survey in this area documented the medicinal plants used for the treatment of cardiometabolic diseases by non-institutionally trained *siddha* practitioners [25], and this survey aimed to document and analyze the medicinal foods prescribed by the non-institutionally trained *siddha* practitioners for prevention or management of various illnesses.

## Methods

### Study area

**Fig. 1:**
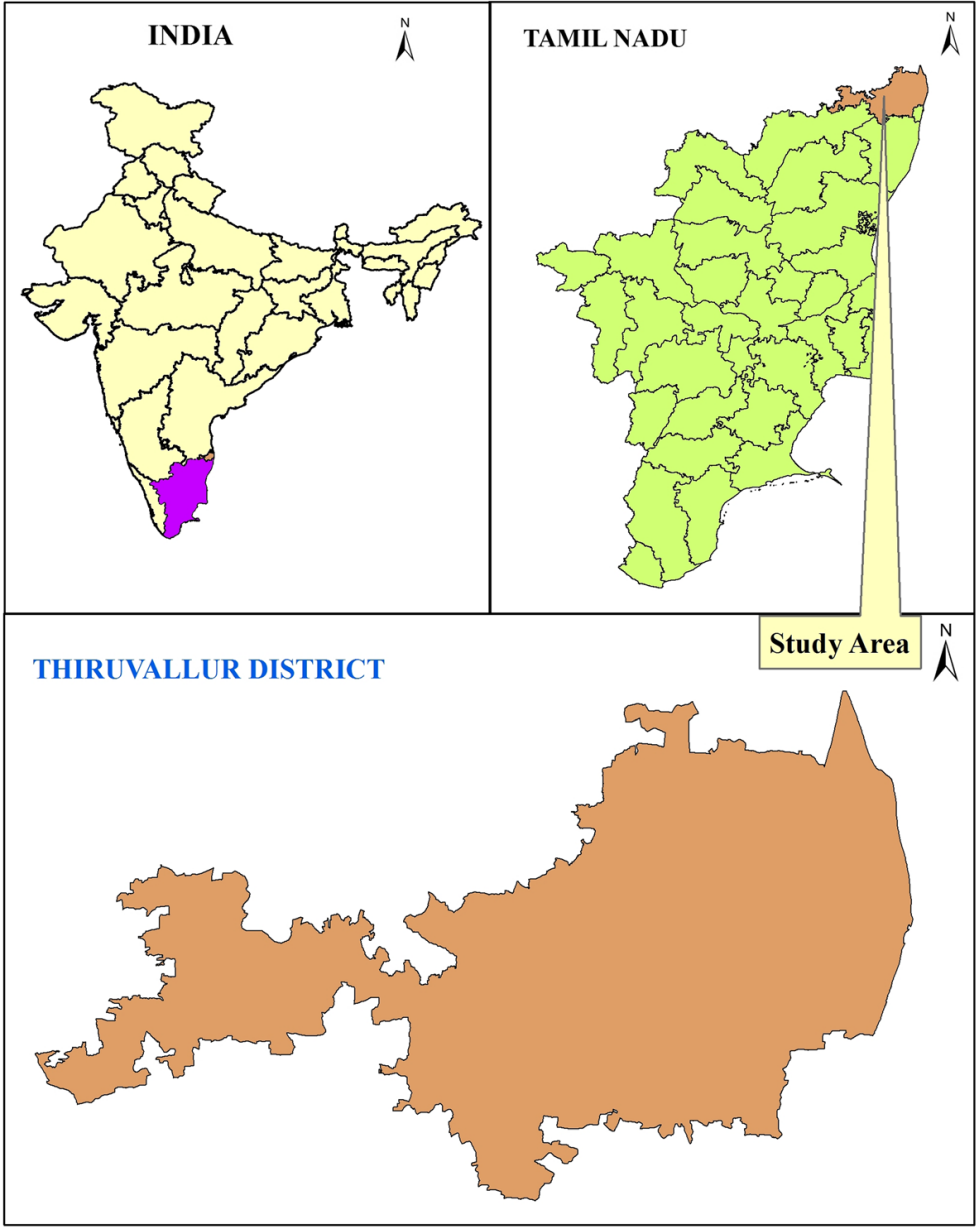
Map showing the location of the study area, Tiruvallur district in Tamil Nadu, India

Tiruvallur district is located in the northern part of Tamil Nadu state between 12°15′–13°15′ N and 79°15′–80°20′ E, with an area of 3423 km^2^ (Fig. 1). The eastern part of this district is bounded by the Bay of Bengal, and the remaining parts are mostly flat and dry. The coastal part of this district occupies 498 km^2^ and has a costal line of 80 km for marine fisheries. The inland fresh water area is 750 km^2^, and brackish water area is 148 km^2^. This district has a forest cover of 197.8 km^2^ which occupies 5.8% of the total area; these forests mostly fall under the classes of dry thorn and dry evergreen. This district has nine *taluks* (sub-districts) and 14 revenue blocks. This district exhibits both urban and rural characteristics. Nearly 47% of the total human workforce deals with agriculture; this district is one of the fastest developing districts of Tamil Nadu in terms of industries. The average rainfall of the district is 1104 mm, out of which 52% is received from northeast monsoon and 41% is received from southwest monsoon. The State Government is providing *siddha* treatment in 12 hospitals with 37 institutionally trained *siddha* practitioners.

### Interviews

The local knowledge of non-institutionally trained *siddha* practitioners on medicinal foods was documented between December 2014 and April 2017 using a questionnaire. The interview protocols used for this survey were in accordance with the previously published methods [26–29] and also with the guidelines of ISE code of ethics for ethnobiological research [30]. This study focused on the local knowledge of *siddha* practitioners who learnt only through traditional methods of teaching (non-institutional training), practicing for a minimum of 5 years, and willing to share their knowledge. Snowball sampling method was used to recruit the informants, and the informants for this survey were recruited irrespective of age, gender, education, and nativity. The aim of this survey was explained to the participants in lay terms: one or two visits were taken to get familiarity, and the formal interviews were conducted after getting written informant consent to participate in this survey. By this way, the local knowledge of 82 non-institutionally trained *siddha* practitioners on medicinal foods was documented in this communication. The protocol used in this survey was approved by the Institutional Ethics Committee for Ethnobiological Research.

The questionnaire used for this survey consisted of two parts. In the first part, the data related to the demography of the informants such as age, gender, education, mode of learning traditional medicine, experience, nativity, residential area, and occupation were documented. In the second part of the questionnaire, the data regarding the medicinal foods that they are prescribing to their patients, the ingredients (plants/animals) used to prepare these foods, parts, mode of preparation, illnesses treated with these foods, dosage, and duration of consumption were documented. Under the ingredients section, the key medicinal taxa which were perceived to attribute the medicinal effect were also documented. Besides it, other details such as the perceived availability of resources (very common - rare), localization of the usage (ubiquity - rare), frequency of usage (frequent - no longer used), parts used, multi-functional usage (different eatable forms reported), taste score (best - terrible), and medicinal role (very high - not recognized) were also documented. The informants were taken to the fields from where they usually collected the samples, including local markets, and asked to show the specimens of the taxa that they mentioned either fresh or in dry condition. The interviews were conducted in the local language *Tamil*, and they were video-graphed. Questionnaires were cross-verified with the video-graphs for ambiguous entries. The data were translated into English in the laboratory. Equivalent English terms for the illnesses were fixed by correlating the Tamil terminologies and symptoms with the biomedical literature by consulting an institutionally trained *siddha* practitioner.

### Specimens

Representative specimens of the fresh plants or crude drugs were collected, herborized, and stored at the museum of Entomology Research Institute, Loyola College, Chennai. The botanical authenticity of the plants was confirmed by the examination of the voucher specimens using local flora [31–34], and the valid names were confirmed with a website [35]. All the animals mentioned in this work were photographed, and their zoological names were confirmed by the Zoologist (MGP), who is one of the authors of this communication.

### Quantification of the data

Medicinal plants/animals which were considered as key medicinal taxa by the informants were taken for the analysis. The illnesses mentioned by the informants were grouped as illness categories on the basis of *emic* perceptions. The illness category *vadha* ailments include the musculoskeletal disorders and *kapha* ailments include the pulmonary and respiratory diseases. The data were then converted into use reports (UR) and claims in accordance with our previous work. Briefly, UR can be described as “informant (*i*) prescribes a species (*s*) for a use category (*u*)” [36]; claims lack the informant (*i*) factor [37]. For example, if two informants mention a species for the treatment of an illness category, it yields two UR and a claim. Sufficiency of sampling of this survey was assessed by plotting the cumulative number of UR and Shannon Wiener’s index, which was calculated using PAST3 program.

Informant consensus over treating illnesses and on the taxa was assessed using informant consensus factor (FIC) and Index of Agreement on Remedies (IAR), respectively, [38] using the following formula.

FIC = (*N*_ur_ − *N*_*t*_)/(*N*_ur_ − 1)

IAR = (*n*_ur_ − *n*_*a*_)/(*n*_*r*_ − 1)

where *N*_ur_ is the number of UR for a particular illness category, *N*_*t*_ is the total number of taxa mentioned for that particular illness category, *n*_ur_ is the total number of UR registered for a taxon, and *n*_*a*_ is the number of illness categories that are treated with that taxon. These factors range from zero to one, where increasing values indicate high rate of informant consensus. Illness categories with high, average, and low consensus were calculated [39, 40]. Local uses of medicinal taxa having high IAR value and UR were compared with global usage pattern and scientific literature.

Humoral properties (cold and hot) of the medicinal plants and their uses in formal *siddha* medicine were taken from *siddha materia medica* [41–44]. If no report was available about a taxon, it was kept under the category “unspecified.” relative frequency of citation (RFC) on the basis of humors for each illness category was calculated using the following formula.

RFC = (number of UR for a humor/total number of UR) ×  100

Cultural Food Significance Index (CFSI) of the key medicinal taxa was calculated in accordance with the method of Pieroni [45] with slight modifications (Table 1). For this calculation, key medicinal taxa with a minimum of two UR were considered. CFSI took seven indices into account, and it was calculated using the following formula.

**Table 1:** Scores for Cultural Food Significance Index

Indices	Attributes	Scores
Availability index (AI)	Availability	
	Very common	4.0
	Common	3.0
	Middle	2.0
	Rare	1.0
	Localization of the use	
	Ubiquity	0.0
	Localized	− 0.5
	Very localized	− 1.0
Frequency of utilization index (FUI)	> Once/week	5.0
	Once/week	4.0
	Once/month	3.0
	> Once/year but < once/month	2.0
	Once/year	1.0
	No longer used during the past 30 years	0.5
Part used index (PUI)	Whole aerial parts	3.0
	Leaves with a few stems, whole aerial parts of very young plants	2.0
	Root/root stocks, bulbs, leaves, fruits	1.5
	Shoots	1.25
	Bark, younger part of roots, stems, leaf stalks, young whorls of leaves, seeds	1.0
	Younger part of shoots, buds, flowers, receptacles	0.75
Multifunctional food use index (MFFI)	Chutney, dosa, gravy, sauce, salad	1.5
	Jam, porridge, pickles, fried, syrups	1.0
	Soup, milk preparation	0.75
	Raw	0.5
Taste Score Appreciation Index (TSAI)	Best	10.0
	Very good	9.0
	Good	7.5
	Fair	6.5
	Poor	5.5
	Terrible	4.0
Food Medicinal Role Index (FMRI)	IAR of the taxa > mean + SD	5.0
	IAR of the taxa < mean + SD but IAR > mean − SD	4.0
	IAR < mean − SD	3.0

CFSI = QI) ×  AI) ×  FUI) ×  PUI) ×  MFFI) ×  TSAI) ×  FMRI ×  10^− 2^

where Quotation index (QI) indicated the number of UR for a taxon. Availability index (AI) was obtained by subtracting scores of localization of the use from the scores of availability. Frequency of use index (FUI), part used index (PUI), multifunctional food use index (MFFI), Taste Score Appreciation Index (TSAI), and Food Medicinal Role Index (FMRI) were calculated as given in Table 1. In the case of FMRI, the taxa with IAR greater than mean plus standard deviation of the IAR of all taxa were given the highest score. The taxa with IAR value lower than mean plus standard deviation of the IAR were given the lowest score. Other taxa were given average FMRI score. For animal taxa, PUI was omitted from calculation.

## Results

### Demographic profile of the informants

Analysis of the informants’ demography indicated that the non-institutionally trained *siddha* tradition is a male dominant domain and a major portion of these practitioners had completed secondary or higher secondary schooling. Considerable portion (36.58%) of the practitioners had migrated to the study area from other districts. It also showed that major portion (71.94%) of practitioners was practicing in urban and semi-urban areas (Table 2).

**Table 2:** Demographic profile of the informants interviewed in the survey (*N* = 82)

	Number	Percent
Age		
35–40	7	8.53
41–50	44	53.65
51–60	26	31.70
Above 60	5	6.09
Gender		
Male	80	97.56
Female	2	2.43
Education		
Primary school	5	6.09
Secondary school	20	24.39
Higher secondary	41	50.00
Degree	16	19.51
Mode of learning		
From family members	13	15.85
From traditional practitioners	69	62.19
Experience		
5 years	1	1.21
6–20 years	62	75.60
21–50 years	19	23.17
Nativity to the district		
Native	52	63.41
Migrated	30	36.58
Residence		
Urban	35	42.68
Semi-urban	24	29.26
Rural	23	28.04
Occupation		
Full time practitioners	82	100

### Descriptive statistics of the medicinal foods

**Fig. 2:**
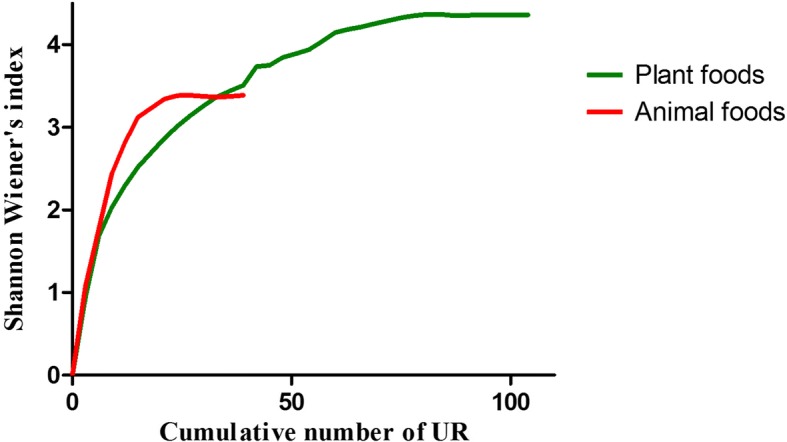
Assessing sampling sufficiency for the plant and animal species cited to prepare medicinal foods by the non-institutionally trained *Siddha* practitioners in Tiruvallur district using Species Accumulation Curve

**Fig. 3:**
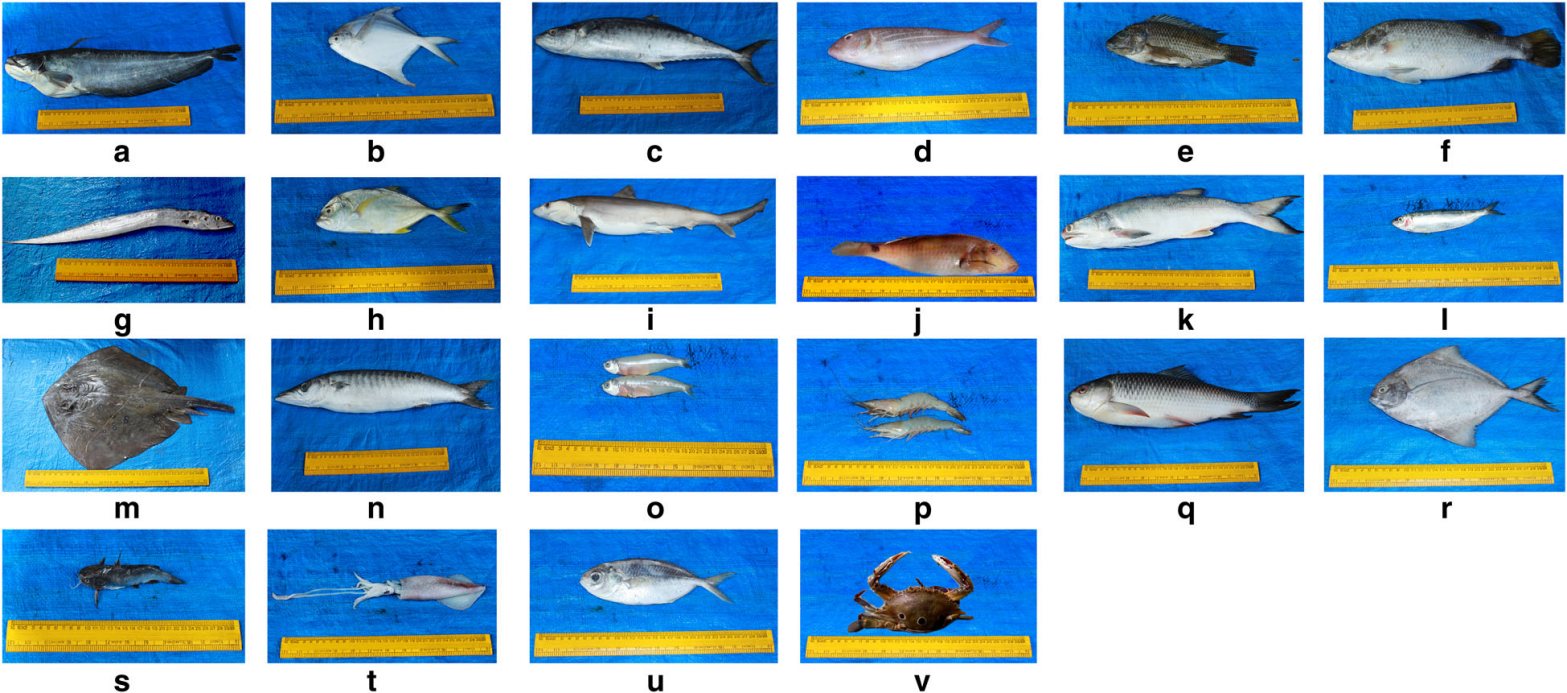
Photographs of some animal taxa referred by the non-institutionally trained *siddha* practitioners of Tiruvallur district for the preparation of medicinal foods. (**a**) Channa sp. (Virāl); (**b**) Pampus argenteus (Vavāl); (**c**) Scomberomorus guttatus (Vañciram); (**d**) Nemipterus japonicas (Caṅkarā); (**e**) Oreochromis mossambicus (Jilēppi); (**f**) Lates calcarifer (Koṭuvā); (**g**) Trichiurus lepturus (Ōlai vāḷai); (**h**) Caranx melampygus (Pāṟai); (**i**) Scoliodon laticaudus (Piḷḷaiccuṟā); (**j**) Parupeneus indicus (Nakarai); (**k**) Eleutheronema tetradactylum (Kālā); (**l**) Sardinella longiceps (Peichālai); (**m**) Himantura uarnak (Tirukkai); (**n**) Sphyraena jello (Ūḻi); (**o**) Stolephorus indicus (Nettili); (**p**) Fenneropenaeus indicus (Veḷḷai iṟāl); (**q**) Lebeo rohita (Kaṇṇādi kendai); (**r**) Parastromateus niger (Karuppu vavāl); (**s**) Mystus sp. (Keḷutti); (**t**) Uroteuthis duvauceli (Ūci kaṉavā); (**u**) Lactarius lactarius (Cutumpu); (**v**) Portunus sanguinolentus (Kaṭal naṇṭu)

This study documented 165 medicinal foods used by the non-institutionally trained *siddha* practitioners of Tiruvallur district to treat various illnesses. Among them 73.93% were plant based, and 26.07% foods used animal taxa as the major key ingredients. To prepare these foods, 104 (72.72%) plant taxa and 39 (27.27%) animal taxa were used and major portion of the animals was represented by fish taxa. Sampling sufficiency analysis showed a clear asymptote of the curve which indicated the sufficiency of the sampling (Fig. 2). Analysis of the data yielded 588 UR and 381 claims. Plant-based formulations gained high number of UR (77.68%) and claims (76.64%). The plant and animal taxa cited by the non-institutionally trained *siddha* practitioners are given in Tables 3 and 4. The medicinal foods prescribed by the informants are given in Additional file 1: Table S1. The photographs of some animal taxa referred by the informants for the preparation of medicinal foods are given in Fig. 3.

**Table 3:** List of plant taxa cited by the non-institutionally trained *siddha* practitioners of Tiruvallur district of Tamil Nadu for preparing medicinal foods

S.No	Binomial name, Voucher number & Family	Vernacular name	Parts used	Humoral property^a^	Reported pharmacological action in *Siddha*^a^	Illnesses treated	Illnesses categories	IAR
1.	*Abelmoschus esculentus* (L.) Moench SE201 (Malvaceae)	*Veṇṭaikkāy*	Tender fruits	Cold	Refrigerant, Aphrodisiac, Demulcent, Diuretic, Emollient, To treat diarrhea and dysentary	Diabetes (2), Coolant (1), Oligospermia (1)	Dia. (2), Coo. (1), Mal. (1)	0.333
2.	*Abutilon indicum* (Link) Sweet SE193 (Malvaceae)	*Tutti*	Leaves	Cold	Demulcent, Tonic, Laxative, Diuretic, Sedative, To treat hemorrhoids, boils, gangrene and warts	Hemorrhoids (3)	Hem. (3)	1.000
3.	*Acalypha indica* L. SE188 (Euphorbiaceae)	*Kuppaimēṉi*	Leaves	Hot	Anodyne, Anthelmintic, Cathartic, Diuretic, Emetic, Expectorant, Emmenagogue, To treat toothache, burns, organic poisons, stomachache, hemorrhoids, joint pain and bronchitis	Bronchitis (1), Hemorrhoids (2), Intestinal worms (1)	Hem. (2), Kap. (1), Gas. (1)	0.333
4.	*Allium cepa* L. SE170 (Amaryllidaceae)	*Veṅkāyam*	Bulbs	Hot	Stimulant, Diuretic, Expectorant, Emmenagogue, Rubefacient, Demulcent, Aphrodisiac, To treat hemorrhoids, eczema, hypertension, mouth ulcers, polydipsia and diarrhea	Hemorroids (1), Bleeding through rectum (3), Oligospermia (2), Burning sensation (1)	Hem. (4), Mal. (2), Coo. (1)	0.666
5.	*Allium sativum* L. SE107 (Amaryllidaceae)	*Veḷḷaippūṇṭu*	Bulbs	Hot	Carminative, Stomahic, Tonic, Alterative, Stimulant, Expectorant, Diuretic, Anthelmintic, To treat boils, cough, wheezing, intestinal worms and hemorrhoids	Indigestion (1), Dysmenorrhea (1), Diabetes (1), Heart ailments (1), Flatulance (1)	Gas. (2), Gyn. (1), Dia. (1), Hea. (1)	0.250
6.	*Aloe vera* (L.) Burm.f. SE108 (Asphodelaceae)	*Kaṟṟāḻai*	Leaves	Cold	Tonic, Alterative, Purgative, Emmenagogue, To treat dermatological ailments, leprosy, hemorrhoids, anal fistula, urolithiasis	Obesity (1), Urolithiasis (1), Liver ailments (1), Dysmenorrhea (2), Metrorrhagia (1), Uteral ailments (1), Gastrointestinal ailments (1)	Gyn. (4), Obe. (1), Uri. (1), Liv. (1), Gas. (1)	0.428
7.	*Alternanthera sessilis* (L.) R.Br. ex DC. SE159 (Amaranthaceae)	*Poṉṉāṅkaṇṇi*	Leaves	Cold	Alterative, Coolant, Good for eyes	Menstrual migraine (1), To increase memory and vision (1)	Gyn. (1), Psy. (1), Eye. (1)	0.000
8.	*Amaranthus viridis* L. SE117 (Amaranthaceae)	*Araikkīrai*	Leaves	Hot	Stimulant, Aphrodisiac	Male infertility (1), Anemia (1), General weakness (2)	Wea. (2), Mal. (1), Blo. (1)	0.333
9.	*Amorphophallus paeoniifolius* (Dennst.) Nicolson SE113 (Araceae)	*Karuṇaikkiḻaṅku*	Rhizomes	Cold	Alterative, Nutritive, Tonic, To treat pulmonary ailments, Hemorrhoids and anorexia	Hemorrhoids (3), Hypertension (1)	Hem. (3), Hpt. (1)	0.666
10.	*Anacardium occidentale* L. SE127 (Anacardiaceae)	*Muntiri*	Seeds	Cold	Tonic, Aphrodisiac	General weakness (2), Fatigue (1)	Wea. (2), Fat. (1)	0.500
11.	*Annona squamosa* L. SE183 (Annonaceae)	*Cītāppaḻam*	Fruits	Cold	Coolant	Coolant (1), Gastric ulcers (1)	Coo. (1), Gas. (1)	0.000
12.	*Arachis hypogaea* L. SE126 (Fabaceae)	*Vērkkaṭalai*	Seeds	Hot	Nutrient, laxative, Emollient	General weakness (2), Fatigue (1)	Wea. (2), Fat. (1)	0.500
13.	*Benincasa hispida* (Thunb.) Cogn. SE200 (Cucurbitaceae)	*Veṇpūcaṇi*	Tender fruits	Cold	Diuretic, Styptic, Tonic, Alterative, Nutrient, To treat dysuria, fever, dermatological ailments, leucorrhea, psychological ailments	Dysuria (1), To gain weight (1)	Uri. (1), Wea. (1)	0.000
14.	*Beta vulgaris* L. SE150 (Amaranthaceae)	*Pīṭrūṭ*	Rhizomes	--	--	Anemia (2), Male infertility (1), Hypotension (1)	Blo. (2), Mal. (1), Hpo. (1)	0.333
15.	*Boerhavia diffusa* L. SE194 (Nyctaginaceae)	*Mūkkiraṭṭai*	Leaves	Hot	Expectorant, Diuretic, Laxative, Coolant, Anthelmintic, Emetic	Dysuria (3)	Uri. (3)	1.000
16.	*Borassus flabellifer* L. SE168 (Arecaceae)	*Paṉai*	Inflorescence	Cold	Coolant, Diuretic	Burning sensation (3), Jaundice (1)	Coo. (3), Jau. (1)	0.666
17.	*Brassica oleracea* var. *gongylodes* L. SE199 Brassicaceae	*Nūkkal*	Stems	--	--	Diabetes (2)	Dia. (2)	1.000
18.	*Cardiospermum halicacabum* L. SE109 (Sapindaceae)	*Muṭakkottāṉ*	Leaves	Hot	Diuretic, Laxative, Stomachic, Rubefacient, Antirheumatic, Nutritive, To treat musculo-skeletal and dermatological ailments	Rhinitis (1), Cough (1), Somatalgia (3)	Ana. (3), Kap. (2)	0.750
19.	*Carica papaya* L. SE182 (Caricaceae)	*Pappāḷi*	Fruits	Hot	Laxative, Tonic, Diuretic, Lactogogue	Constipation (1), Lactogogue (1), Joint pain (1)	Gas. (1), Gyn. (1), Vad. (1)	0.000
20.	*Cassia fistula* L. SE197 (Fabaceae)	*Carakkoṉṟai*	Flowers	Hot	Vermifuge, To treat leucorrhea, anemia, jaundice, dermatological infections and diabetes	Diabetes (1)	Dia. (1)	0.000
21.	*Centella asiatica* (L.) Urban SE157 (Apiaceae)	*Vallārai*	Leaves	Cold	Alterative, Tonic, Diuretic, Stimulant, Emmenagogue, To treat fever, elephantiasis, scrotal swelling, Swollen lymph nodes, eczema, wounds and swellings	To strengthen memory (2), Hypothyroidism (1)	Psy. (2), Thy. (1)	0.500
22.	*Cicer arietinum* L. SE129 (Fabaceae)	*Koṇṭaikkaṭalai*	Seeds	Cold	Antibilious, Diuretic, Aphrodisiac	General weakness (2), Fatigue (1), Menstrual cramps (1)	Wea. (2), Fat. (1), Gyn. (1)	0.333
23.	*Cichorium intybus* L. SE176 (Asteraceae)	*Kāciṉikkīrai*	Leaves	--	--	Male infertility (1)	Mal. (1)	0.000
24.	*Cissus quadrangularis* L. SE137 (Vitaceae)	*Piraṇṭai*	Stem	Hot	Alterative, Emmenagogue, Stomachic, To treat hemorrhoids, anorexia, ulcers, diarrhea and fatigue	Obesity (1), Gastric ulcers (1), Bone fractures (1), Hemorrhoids (3)	Hem. (3), Obe. (1), Gas. (1), Bon. (1)	0.400
25.	*Citrullus lanatus* (Thunb.) Matsum. & Nakai SE178 (Cucurbitaceae)	*Tarpūcaṇi*	Fruits	Cold	Coolant, Diuretic	Hyperacidity (2), Dysuria (1)	Gas. (2), Uri. (1)	0.500
26.	*Citrus limon* (L.) Osbeck SE167 (Rutaceae)	*Elumiccai*	Fruits	Hot	Carminative, Rubefacient, To treat fainting, nausea, polydipsia, psychological ailments, eye ailments	Indigestion (3), Constipation (1), Heart ailments (1), Acne (1), Diabetes (1), Arthritis (2), To increase vision (1), Hypothyroidism (1)	Gas. (4), Vad. (2), Hea. (1), Der. (1), Dia. (1), Eye. (1), Thy. (1)	0.400
27.	*Citrus medica* L. SE184 (Rutaceae)	*Nārattai*	Fruits	Cold	Aromatic, Stomachic, Astringent, Sedative, Digestive, Good to treat hypertension	Hyperacidity (1), Heart ailments (2), Gastric ulcers (2), Hypertension (2), Anorexia (2), Dermatological ailments (1)	Gas. (5), Hea. (2), Hpt. (2), Der. (1)	0.666
28.	*Citrus reticulata* Blanco SE185 (Rutaceae)	*Ārañcu*	Fruits	--	--	Heart ailments (1)	Hea. (1)	0.000
29.	*Cleome gynandra* L. SE190 (Cleomaceae)	*Nalla vēḷai*	Leaves	Hot	Rubefacient, Anthelmintic, Antispasmodic, Carminative, Diaphoretic	Joint pain (1), Rhinitis (2), Fever (1), Heart ailments (1)	Kap. (2), Fev. (1), Vad. (1), Hea. (1)	0.250
30.	*Coccinia grandis* (L.) Voigt SE147 (Cucurbitaceae)	*Kōvaikkāy*	Tender fruits	Cold	Expectorant, Antispasmodic, Febrifuge, To treat anorexia, fever, bronchitis and eczema	Gastric ulcers (3), Mouth ulcers (2), Oliguria (1), Diabetes (5), Burning sensation (2), Bronchitis (1)	Gas. (5), Dia. (5), Coo. (2), Uri. (1), Kap. (1)	0.692
31.	*Cocculus hirsutus* (L.) Diels SE139 (Menispermaceae)	*Kaṭṭukkoṭi*	Leaves	Hot	Alterative, Laxative, Demulcent, Coolant, To treat diarrhea, metrorrhagia, dysuria and gastric ulcers	Oligospermia (1), Coolant (1)	Mal. (1), Coo. (1)	0.000
32.	*Cocos nucifera* L. SE154 (Arecaceae)	*Tēṅkāy*	Endosperm	Cold	Coolant, Aperient, Nutrient, Diuretic, To treat mouth ulcers	Male infertility (1), Hypotension (1), Gastric ulcers (1), Mouth ulcers (1)	Gas. (2), Mal. (1), Hpo. (1)	0.333
33.	*Cucumis sativus* L. SE174 (Cucurbitaceae)	*Veḷḷarikkāy*	Tender fruits	Cold	Diuretic, Nutrient, Demulcent, Coolant	Hyperacidity (2)	Gas. (2)	0.000
34.	*Cuminum cyminum* L. SE134 (Apiaceae)	*Cīrakam*	Seeds	Cold	Carminative, Stimulant, Stomachic, Astringent, To treat hypertension, liver ailments, urolithiasis, dysentery, wheezing, rhinitis, insomnia	Somatalgia (1), Anemia (1), Anorexia (1), Coolant (2), Hypertension (1), Gastric ulcers (2)	Gas. (3), Coo. (2), Ana. (1), Blo. (1), Hpt. (1)	0.428
35.	*Daucus carota* L. SE153 (Apiaceae)	*Kāraṭ*	Rhizome	--	--	Male infertility (1), Hypotension (1), Bloating (1), Nausea (1)	Gas. (2), Mal. (1), Hpo. (1)	0.333
36.	*Digera muricata* (L.) Mart. SE192 (Amaranthaceae)	*Toyyakkīrai*	Leaves	Cold	Coolant	Diarrhea (1), Dysentery (1)	Gas. (2)	1.000
37.	*Echinochloa frumentacea* Link SE123 (Poaceae)	*Kutiraivāli*	Seeds	--	--	General weakness (2), Fatigue (1)	Wea. (2), Fat. (1)	0.500
38.	*Eclipta prostrata* (L.) L. SE177 (Asteraceae)	*Karicalāṅkaṇṇi*	Leaves	Hot	Cholagogue, Tonic, Alterative, Emetic, Laxative, Deobstruent, Hepatoprotective, To treat throat ailments, jaundice, leprosy, ascites, anemia and toothache	To increase memory (1) and vision (1), Liver ailments (2), Anemia (2)	Liv. (2), Blo. (2), Psy. (1), Eye. (1)	0.400
39.	*Eleusine coracana* Gaertn. SE118 (Poaceae)	*Kēḻvaraku*	Seeds	Cold	Nutrient, Astringent, To treat diabetes	General weakness (2), Fatigue (1), Diabetes (1), To strengthen memory (1), Anemia (2)	Wea. (2), Blo. (2), Fat. (1), Dia. (1), Psy. (1)	0.333
40.	*Erythrina variegata* L. SE138 (Fabaceae)	*Kalyāṇamuruṅkai*	Leaves	Hot	Diuretic, Laxative, Emmenagogue, Lactogogue	Bronchitis (1)	Kap. (1)	0.000
41.	*Ferula assa-foetida* L. SE136 (Apiaceae)	*Peruṅkāyam*	Resin	Hot	Stimulant, Carminative, Antispasmodic, Expectorant, Laxative, Anthelmintic, Diuretic, Aphrodisiac, Emmenagogue	Somatalgia (1)	Ana. (1)	0.000
42.	*Ficus benghalensis* L. SE142 (Moraceae)	*Āl*	Fruits	Cold	Astringent, Tonic, Aphrodisiac	Oligospermia (1)	Mal. (1)	0.000
43.	*Ficus racemosa* L. SE140 (Moraceae)	*Atti*	Fruits	Cold	Astringent, Laxative, Good to treat diarrhea, hemorrhoids and anemia	Oligospermia (1), Anemia (2), Male infertility (1), Constipation (1), Wheezing (1), Gastric ulcers (1), Hemorrhoids (1)	Mal. (2), Blo. (2), Gas. (2), Hem. (1), Kap. (1)	0.428
44.	*Ficus religiosa* L. SE141 (Moraceae)	*Aracu*	Fruits	Cold	Laxative, Coolant	Oligospermia (1)	Mal. (1)	0.000
45.	*Foeniculum vulgare* Mill. SE114 (Apiaceae)	*Peruñcīrakam*	Seeds	Hot	Carminative, Stomachic, To treat uteral ailments, fever, indigestion, bloating, cough, liver ailments, wheezing, rhinitis	Heart ailments (1)	Hea. (1)	0.000
46.	*Garcinia gummi-gutta* (L.) Roxb. SE101 (Clusiaceae)	*Koṭampuḷi*	Fruits	Hot	Carminative, Digestive	Obesity (1)	Obe. (1)	0.000
47.	*Gossypium* spp*.* SE169 (Malvaceae)	*Parutti*	Seeds	Hot	Laxative, Expectorant, Aphrodisiac	General weakness (1), Somatalgia (1)	Wea. (1), Ana. (1)	0.000
48.	*Hibiscus cannabinus* L. SE202 (Malvaceae)	*Puḷiccakīrai*	Leaves	Hot	Emollient, Laxative, To treat anorexia and hypertension	Anorexia (1)	Gas. (1)	0.000
49.	*Hibiscus rosa-sinensis* L. SE102 (Malvaceae)	*Cemparattai*	Flowers	Cold	Laxative, Aphrodisiac, Emmenagogue, Emollient, Demulcent, Coolant, To treat leucorrhea, metrorrhagia and hypertension	Male infertility (1), Heart ailments (2)	Hea. (2), Mal. (1)	0.500
50.	*Hybanthus enneaspermus* (G.Don) R.Br. ex Arn. SE162 (Violaceae)	*Oritaḻtāmarai*	Flowers	Cold	Nutritive, Aphrodisiac	Oligospermia (1), Hypothyroidism (1)	Mal. (1), Hpo. (1)	0.000
51.	*Ipomoea aquatica* Forssk*.* SE191 (convolvulaceae)	*Vaḷḷaikkīrai*	Leaves	Cold	Coolant, Lactogogue, Aphrodisiac, Antidiabetic	Mouth ulcers (1), Gastric ulcers (2), Oligospermia (2)	Gas. (3), Mal. (2)	0.750
52.	*Lagenaria siceraria* (Molina) Standl. SE104 (Cucurbitaceae)	*Curaikkāy*	Tender fruits	Cold	Coolant, Diuretic, Nutritive, Antibilious	Obesity (1), Coolant (1), To strengthen memory (1), Swelling of the limbs (1), Dysuria (2)	Uri. (3), Obe. (1), Coo. (1), Psy. (1)	0.500
53.	*Leucas aspera* (Willd.) Link SE112 (Lamiaceae)	*Tumpai*	Leaves	Hot	Laxative, Expectorant, Stimulant, Emmenagogue, To treat headache, throat ailments, polydipsia, cough, bronchitis, sinusitis, leucorrhea, fatigue, somatalgia	Rhinitis (1), Cough (1), Somatalgia (1)	Kap. (2), Ana. (1)	0.500
54.	*Limonia acidissima* Groff SE148 (Rutaceae)	*Viḷā*	Fruit bulbs	Cold	Aromatic, Coolant, To treat anorexia, bloating, polydipsia, cough and bronchitis	Diabetes (3), Polydipsia (1), Hypertension (1)	Dia. (4), Hpt. (1)	0.750
55.	*Macrotyloma uniflorum* (Lam.) Verdc. SE100 (Fabaceae)	*Koḷḷu*	Seeds	Hot	Astringent, Diuretic, Tonic	Obesity (1)	Obe. (1)	0.000
56.	*Mangifera indica* L. SE181 (Anacardiaceae)	*Mā*	Fruits	Hot	Laxative, Diuretic, Tonic, Aphrodisiac	Male infertility (2), Heart ailments (4), Anorexia (3), To increase vision (1)	Hea. (4), Mal. (2), Gas. (3), Eye. (1)	0.666
57.	*Marsilea quadrifolia* L. SE195 (Marsileaceae)	*Āraikkīrai*	Leaves	Cold	Coolant, Polyuria	Polyuria (2), Ulcers in urinary tract (2)	Dia. (2), Uri. (2)	0.666
58.	*Melochia corchorifolia* L. SE189 (Malvaceae)	*Puṇṇākkukkīrai*	Leaves	Cold	Diuretic, Laxative	Pain during menopause (1)	Gyn. (1)	0.000
59.	*Mentha arvensis* L. SE171 (Lamiaceae)	*Putiṉā*	Leaves	Hot	Stomachic, Diuretic, Stimulant, Carminative, Antispasmodic	Anemia (1), Anorexia (1)	Blo. (1), Ana. (1)	0.000
60.	*Momordica charantia* L. SE149 (Cucurbitaceae)	*Pākaṟkāy*	Tender fruits	Hot	Tonic, Stomachic, Stimulant, Antibilious, Laxative, Alterative, Anthelmintic	Diabetes (1), Bloating (1), Intestinal worms (1)	Gas. (2), Dia. (1)	0.500
61.	*Moringa oleifera* Lam. SE111 (Moringaceae)	*Muruṅkai*	Leaves	Cold	Antispasmodic, Stimulant, Expectorant, Diuretic, To treat anorexia, headache, fainting and eye ailments	Rhinitis (1), Cough (1), Somatalgia (2), Anemia (5), General weakness (1), To increase vision (1), Hypertension (2), Diabetes (1), Obesity (1), Anorexia (1), Menstrual migraine (2)	Blo. (5), Ana. (2), Hpt. (2), Gyn. (2), Kap. (2), Wea. (1), Eye. (1), Dia. (1), Obe. (1), Gas. (1)	0.470
62.	*Mukia maderaspatana* (L.) M.Roem. SE160 (Cucurbitaceae)	*Mucumucukkai*	Leaves	Hot	Expectorant, To treat cough, bronchitis, wheezing, rhinitis	Asthma (2), Bronchitis (2), Cough (1)	Kap. (5)	1.000
63.	*Murraya koenigii* (L.) Sprengel SE106 (Rutaceae)	*Kaṟivēppilai*	Leaves	Hot	Tonic, Stomachic, Diarrhea, Nausea, Fever, Psychological ailments	Indigestion (2), Dysmenorrhea (1), Diabetes (1), Anemia (1), Obesity (1), Anorexia (1), Bloating (1)	Gas. (4), Gyn. (1), Dia. (1), Blo. (1), Obe. (1)	0.428
64.	*Musa paradisiaca* L. SE145 (Musaceae)	*Vāḻai*	Tender fruits, Flowers	Hot	Demulcent, Laxative, Nutritive, To treat hemorrhoids	Menstrual cramps (3), Gastric ulcers (3), Mouth ulcers (1), Hyperacidity (1), Hemorrhoids (2), Bleeding through rectum (1)	Gas. (5), Hem. (3), Gyn. (3)	0.800
65.	*Nelumbo nucifera* Gaertn. SE105 (Nelumbonaceae)	*Tāmarai*	Flowers	Cold	Coolant, Astringent, Expectorant, Sedative, To treat fever, polydipsia and liver ailments	Heart ailments (1)	Hea. (1)	0.000
66.	*Nigella sativa* L. SE135 (Ranunculaceae)	*Karuñcīrakam*	Seeds	Hot	Carminative, Diuretic, Emmenagogue, Lactogogue, Anthelmintic, Stomachic, Antibiotic, Emmollient, To treat eczema, headache, cough, vomiting, nausea and jaundice	Somatalgia (1), Amenorrhea (1)	Ana. (1), Gyn. (1)	0.000
67.	*Oldenlandia umbellata* L. SE161 (Rubiaceae)	*Impūral*	Leaves	Cold	Expectorant, Styptic, Cholagogue, Good to treat internal bleeding	Productive cough (1), Bronchitis (1), Sinusitis (1)	Kap. (3)	1.000
68.	*Oryza sativa* L. SE130 (Poaceae)	*Arici*	Seeds	Cold	Nutrient, Demulcent, Coolant	General weakness (2), Fatigue (1), Diabetes (1), Anemia (1)	Wea. (2), Fat. (1), Dia. (1), Blo. (1)	0.250
69.	*Oxalis corniculata* L. SE203 (Oxalidaceae)	*Puḷiyārai*	Leaves	Cold	Stomachic, Coolant, Astringent, To treat fainting, diarrhea, bleeding through anus and hemorrhoids	Hypertension (2), Insomnia (1)	Hpt. (2), Psy. (1)	0.500
70.	*Panicum sumatrense* Roth ex Roem. & Schult. SE121 (Poaceae)	*Cāmai*	Seeds	Cold	Demulcent, Tonic, To treat polydipsia, fever and musculo-skeletal disorders	General weakness (2), Fatigue (1)	Wea. (2), Fat. (1)	0.500
71.	*Paspalum scrobiculatum* L. SE120 (Poaceae)	*Varaku*	Seeds	Cold	Chologogue	General weakness (2), Fatigue (1)	Wea. (2), Fat. (1)	0.500
72.	*Pennisetum glaucum* (L.)R.Br. SE119 (Poaceae)	*Kampu*	Seeds	Cold	Tonic	General weakness (2), Fatigue (1), Anemia (1)	Wea. (2), Fat. (1), Blo. (1)	0.333
73.	*Phoenix dactylifera* L. SE156 (Arecaceae)	*Pērīṭcai*	Fruits	Hot	Tonic, Nutritive, Demulcent, Laxative, Diuretic, Febrifuge, Coolant, Expectorant, Aphrodisiac, Good to treat polydipsia, anorexia and diabetes	Male infertility (1), Hypotension (1), General weakness (1)	Mal. (1), Hpo. (1), Wea. (1)	0.000
74.	*Phyllanthus emblica* L. SE152 (Phyllanthaceae)	*Nellikkāy*	Fruits	Cold	Astringent, Coolant, Diuretic, Laxative, To treat bronchitis, sinusitis, nausea, vomiting, giddiness and hypertension	Heart ailments (2), Anemia (2), Constipation (1), Wheezing (1), Burning sensation (1), Diabetes (1), Anorexia (2), Rheumatalgia (1), To increase vision (2), Hypothyroidism (1)	Gas. (3), Hea. (2), Blo. (2), Eye. (2), Kap. (1), Coo. (1), Dia. (1), Vad. (1), Hpo. (1)	0.384
75.	*Piper longum* L. SE133 (Piperaceae)	*Tippili*	Seeds	Hot	Stimulant, Carminative, To treat cough, gastric ulcers, wheezing, anemia, fainting, anorexia, bloating, headache, sinusitis, throat ailments and oligospermia	Somatalgia (1)	Ana. (1)	0.000
76.	*Piper nigrum* L. SE132 (Piperaceae)	*Miḷaku*	Seeds	Hot	Acrid, Carminative, Febrifuge, Rubefacient, Stimulant, Resolvent, Antidote	Somatalgia (1), Anemia (1), Anorexia (1)	Ana. (1), Blo. (1), Gas. (1)	0.000
77.	*Plectranthus amboinicus* (Lour.) Spreng. SE143 (Lamiaceae)	*Ōmavalli*	Leaves	Hot	Stimulant, Diaphoretic, Expectorant	Rhinitis (1), Bronchitis (1)	Kap. (2)	1.000
78.	*Portulaca quadrifida* L. SE173 (Portulacaceae)	*Ciṟu pacalai*	Leaves	Cold	Diuretic, Stomachic, Aphrodisiac, Antibilious	Dysuria (2), Gastric ulcers (2), Hypertension (1), Anemia (1)	Uri. (2), Gas. (2), Hpt. (1), Blo. (1)	0.400
79.	*Prunus dulcis* (Mill.) D. A. Webb SE128 (Rosaceae)	*Vātumai*	Seeds	Cold	Demulcent, Emollient, Nutrient	General weakness (2), Fatigue (1)	Wea. (2), Fat. (1)	0.500
80.	*Psidium guajava* L. SE179 (Myrtaceae)	*Koyyā*	Fruits	Hot	Tonic, Astringent	Hyperacidity (1), Constipation (2), Diabetes (1)	Gas. (3), Dia. (1)	0.666
81.	*Punica granatum* L. SE166 (Lythraceae)	*Mātuḷai*	Fruits	Cold	Astringent, Coolant	Bromhirdosis (1), Hyperacidity (1), Constipation (1), Gastric ulcers (1), Anorexia (2), Wheezing (1), Rheumatalgia (1), To increase vision (1), Hypothyroidism (1)	Gas. (5), Der. (1), Kap. (1), Vad. (1), Eye. (1), Thy. (1)	0.444
82.	*Senna auriculata* (L.) Roxb. SE103 (Fabaceae)	*Āvārai*	Flowers	Cold	Astringent, Tonic, Good to treat Diabetes	Male infertility (1), Lumbago (1), Diabetes (2), Burning sensation (1)	Mal. (1), Vad. (1), Dia. (2), Coo. (1)	0.000
83.	*Sesamum indicum* L. SE116 Pedaliaceae	*Eḷ*	Seeds	Hot	Emmenagogue, Stimulant, Tonic, Diuretic, Lactogogue, Laxative	Male infertility (1), Diabetes (1), Amenorrhea (1)	Mal. (1), Dia. (1), Gyn. (1)	0.000
84.	*Sesbania grandiflora* (L.) Poiret SE165 (Fabaceae)	*Akatti*	Leaves	Cold	Antidote, Coolant, Laxative, Vermifuge	Obesity (1), Gastric ulcers (3), Hemorrhoids (1), Burning sensation (1), Dysuria (1), Hypertension (1)	Gas. (3), Obe. (1), Hem. (1), Coo. (1), Uri. (1), Hpt. (1)	0.285
85.	*Setaria italica* (L.) P. Beauvois SE122 (Poaceae)	*Tiṉai*	Seeds	Hot	Nutrient, Diuretic, Astringent, Appitizer	General weakness (2), Fatigue (1)	Wea. (2), Fat. (1)	0.500
86.	*Solanum americanum* Mill. SE158 (Solanaceae)	*Maṇattakkāḷi*	Leaves	Cold	Alterative, Diuretic, Diaphoretic, Expectorant, To treat mouth ulcers and bronchitis	Menstrual migraine (1), Strength bones (1) and nerves (1), Bronchitis (1), Somatalgia (2), Heart ailments (1), Wheezing (1), Gastric ulcers (2), Mouth ulcers (2), Fever (1), Fatigue (1), Convulsions (1), Headache (1), Liver diseases (1), Jaundice (1), Mumps (1), Cough (1), Dermatological ailments (1), Hypertension (1), Constipation (1)	Gas. (5), Kap. (3), Der. (2), Neu. (2), Ana. (2), Gyn. (1), Bon. (1), Hea. (1), Fev. (1), Fat. (1), Hed. (1), Liv. (1), Jau. (1), Hpt. (1)	0.409
87.	*Solanum lycopersicum* L. SE180 (Solanaceae)	*Takkāḷi*	Fruits	Cold	Tonic, Coolant, To treat anemia	Constipation (1), Heart ailments (1), Diabetes (1), Anorexia (1), Rheumatalgia (1), To increase vision (1)	Gas. (2), Hea. (1), Dia. (1), Vad. (1), Eye. (1)	0.200
88.	*Solanum torvum* Sw. SE146 (Solanaceae)	*Cuṇṭaikkāy*	Dried fruits	Hot	Expectorant, Germicide, Stomachic, To treat bloating, bronchitis, anorexia, intestinal worms and indigestion	Hypotension (1), Heart ailments (1), Bronchitis (1), Anemia (1), Wheezing (1), Diarrhea (1), Diabetes (1)	Kap. (2), Hpo. (1), Hea. (1), Blo. (1), Gas. (1), Dia. (1)	0.166
89.	*Solanum trilobatum* L. SE110 (Solanaceae)	*Tūtuvaḷai*	Leaves	Hot	Stimulant, Expectorant, Tonic, Aphrodisiac, To treat bronchitis, cough and rhinitis	Rhinitis (1), Cough (1), Somatalgia (1), To increase memory (1), Male infertility (2), Oligospermia (1)	Mal. (3), Kap. (2), Ana. (1), Psy. (1)	0.500
90.	*Sorghum bicolor* (L.) Moench SE124 (Poaceae)	*Veḷḷaiccōḷam*	Seeds	Cold	Nutrient, Laxative	General weakness (2), Fatigue (1)	Wea. (2), Fat. (1)	0.500
91.	*Spinacia oleracea* L. SE175 Amaranthaceae	*Pālakkīrai*	Leaves	--	--	Hypertension (1)	Hpt. (1)	0.000
92.	*Syzygium cumini* (L.) Skeels SE151 (Myrtaceae)	*Nāval*	Fruits	Cold	Stomachic, Diuretic, Tonic, Astringent, coolant, To treat polyuria, polydipsia and dysuria	Diabetes (2)	Dia. (2)	1.000
93.	*Tamarindus indica* L. SE198 (Fabaceae)	*Puḷi*	Fruits	Hot	Laxative, To treat vomiting and hypertension	Anorexia (1), Nausea (1)	Gas. (2)	1.000
94.	*Trachyspermum ammi* Sprague. SE115 (Apiaceae)	*Ōmam*	Seeds	Hot	Stomachic, Antispasmodic, Carminative, Antiseptic, Stimulant, Tonic, Sialogogue, to treat Cough, Diarrhea, Wheezing and Toothache	Heart ailments (1), Somatalgia (1)	Hea. (1), Ana. (1)	0.000
95.	*Trianthema portulacastrum* L. SE187 (Aizoaceae)	*Cāraṇai*	Leaves	Hot	Laxative, Diuretic, To treat jaundice, swelling, anemia and bronchitis	Heart ailments (1)	Hea. (1)	0.000
96.	*Tribulus terrestris* L. SE196 (Zygophyllaceae)	*Ciṟu neruñcil*	Leaves	Cold	Coolant, Diuretic, Demulcent, Tonic, Aphrodisiac, Astringent, To treat dysuria, fever, urolithiasis, enlargement of prostate, leucorrhea and polydipsia	Dysuria (2), Oligospermia (2), Burning sensation (1)	Uri. (2), Mal. (2), Coo. (1)	0.500
97.	*Trichosanthes cucumerina* L. SE172 (Cucurbitaceae)	*Puṭal*	Tender fruits	Cold	Coolant, Aphrodisiac	Oliguria (1)	Uri. (1)	0.000
98.	*Trigonella foenum-graecum* L. SE163 (Fabaceae)	*Ventayam*	Seeds	Cold	Coolant, Laxative, Diuretic, Demulcent, Astringent, Emollient, Aphrodisiac, Carminative, Tonic, To treat diarrhea, burning sensation, hypertension, fever, polydipsia and cough	Diabetes (1), Burning sensation (1), Anemia (2)	Blo. (2), Dia. (1), Coo. (1)	0.333
99.	*Triticum aestivum* L. SE164 (Poaceae)	*Kōtumai*	Seeds	Cold	Nutritive, Demulcent, Aphrodisiac, Antirheumatic	Diabetes (1)	Dia. (1)	0.000
100.	*Vigna mungo* (L.) Hepper SE144 (Fabaceae)	*Uḷuntu*	Seeds	Cold	Demulcent, Coolant, Aphrodisiac, Lactogogue, Nervine tonic, Nutritive, Good to strengthen pelvic bones	General weakness (3), Burning sensation (1), Cervicalgia (1), Lumbago (1), Ovulation problems (1), As supplement after puberty (1), Diabetes (1), Anemia (1)	Wea. (3), Vad. (2), Gyn. (2), Coo. (1), Dia. (1), Blo. (1)	0.444
101.	*Vitis vinifera* L. SE155 (Vitaceae)	*Tirāṭcai*	Fruits	Cold	Laxative, Coolant, Diuretic, Nutritive, to treat anemia, bleeding and heart ailments	Male infertility (1), Hypotension (1), Anemia (2), Anorexia (2), Constipation (1), Wheezing (1), Rheumatalgia (1)	Gas. (3), Blo. (2), Mal. (1), Hpo. (1), Kap. (1), Vad. (1)	0.375
102.	*Zea mays* L. SE125 (Poaceae)	*Makkāccōḷam*	Seeds	Cold	Tonic, Aphrodisiac	General weakness (2), Fatigue (1)	Wea. (2), Fat. (1)	0.500
103.	*Zingiber officinale* Roscoe. SE131 (Zingiberaceae)	*Iñci*	Rhizomes	Hot	Carminative, Stomachic, Sialogogue, Digestive, Stimulant, Rubefacient	Somatalgia (1), Bloating (1)	Ana. (1), Gas. (1)	0.000
104.	*Ziziphus jujuba* Mill. SE186 (Rhamnaceae)	*Ilantai*	Fruits	Cold	Astringent, Emollient, Appitizer, Antiemetic	Anorexia (3), Nausea (1), Diabetes (1)	Gas. (4), Dia. (1)	0.750

^**a**^ - Data taken from *siddha materia medica* [41, 42]; Values given with in the parentheses indicate the number of UR for the respective illness/illness category

**Table 4:** List of animal taxa cited by the non-institutionally trained *siddha* practitioners of Tiruvallur district of Tamil Nadu for preparing medicinal foods

S.No	Binomial name	Vernacular name	Parts used	Humoral property^a^	Reported pharmacological action in *Siddha*^a^	Illnesses treated	Illnesses categories	IAR
1.	*Anguilla bengalensis bengalensis* (J. E. Gray, 1831)	*Vilāṅku*	Meat	Cold	Aphrodisiac	Fatigue (1), Joint pain (1)	Fat. (1). Vad. (1)	0.000
2.	*Bos taurus* Linnaeus, 1758	*Pacu*	Milk	Cold	To treat fever, internal ulcers, pain, urinary ailments, fatigue and emaciation	To increase memory (1), Burning sensation (2), Oliguria (1), Indigestion (1)	Psy. (1), Coo. (2), Uri. (1), Gas. (1)	0.500
3.	*Capra aegagrus hircus* (Linnaeus, 1758)	*Veḷḷāṭu*	Meat	Cold	Nutritive, Aphrodisiac, To treat fever and tuberculosis	Oligospermia (1), Impotence (1), Gastric ulcers (1), Bone fractures (2)	Mal. (2), Gas. (1), Bon. (2)	0.500
4.	*Caranx melampygus* Cuvier, 1833	*Pāṟai*	Meat	--	--	Anabolic (1), Cough (1), Chest pain (1), Wheezing (1), Coolant (1)	Anb. (1), Kap. (2), Hea. (1), Coo. (1)	0.250
5.	*Channa* spp.	*Virāl*	Meat	--	--	Anabolic (1), Oligospermia (1), Coolant (1)	Anb. (1), Mal. (1), Coo. (1)	0.000
6.	*Chanos chanos* (Forsskal, 1775)	*Pāl keṇṭai*	Meat	Cold	--	Lactogogue (1), Fatigue (1), Impotence (1)	Gyn. (1), Fat. (1), Mal.(1)	0.000
7.	*Eleutheronema tetradactylum* (Shaw, 1804)	*Kālā*	Meat	--	--	Impotence (1), Heart diseases (1), Hypertension (1), Urolithiasis (2)	Uri. (2), Mal. (1), Hea. (1), Hpt. (1)	0.250
8.	*Euthynnus affinis* (Cantor, 1849)	*Nīla tuṭuppu cūṟai*	Meat	--	--	Fever (1), Cough (2)	Kap. (2), Fev. (1)	0.500
9.	*Fenneropenaeus indicus* (Milne-Edwards, 1837)	*Veḷḷai iṟāl*	Meat	Hot	Anabolic, To treat anemia	To improve vision (1), Anabolic (3), Lactogogue (1)	Anb. (3), Eye. (1), Gyn. (1)	0.500
10	*Gallus gallus domesticus* (Linnaeus, 1758)	*Kōḻi*	Eggs	Cold	Antirhematic, Aphrodisiac, To treat ulcers and bronchitis	Anemia (2), Bronchitis for children (1)	Blo. (2), Kap. (1)	0.500
11.	*Gerres oyena* (Forsskal, 1775)	*Uṭuvāṉ*	Meat	--	--	Somatalgia (1), Bronchitis (1), Lactogogue (1)	Anb. (1), Kap. (1), Gyn. (1)	0.000
12.	*Gibelion catla* (Hamilton, 1822)	*Kaṭlā*	Meat	Cold	--	Fatigue (2), Somatalgia (1), Impotence (1), Lactogogue (2), To increase vision (1)	Fat. (2), Gyn. (2), Ana. (1), Mal. (1), Eye. (1)	0.333
13.	*Himantura uarnak* (Forsskal, 1775)	*Tirukkai*	Meat	Hot	Aphrodisiac	Somatalgia (1), Wheezing (3)	Kap. (3), Ana. (1)	0.666
14.	*Katelysia opima* (Gmelin, 1791)	*Cippi*	Meat	--	--	Bronchitis (1), Impotence (2)	Mal. (2), Kap. (1)	0.500
15.	*Lactarius lactarius* (Bloch & Schneider, 1801)	*Cutumpu*	Meat	--	--	Chest pain (1)	Hea. (1)	0.000
16.	*Lates calcarifer* (Bloch, 1790)	*Koṭuvā*	Meat	--	--	Rheumatalgia (3), Impotence (1)	Vad. (3), Mal. (1)	0.666
17.	*Lebeo rohita* Hamilton, 1822	*Kaṇṇādi kendai*	Meat	--	--	Somatalgia (1), Heart disease (1), Eye disease (1), Obesity (1)	Ana. (1), Hea. (1), Eye. (1), Obe. (1)	0.000
18.	*Monodactylus argenteus* (Linnaeus, 1758)	*Puraṇṭi*	Meat	--	--	Cough (1)	Kap. (1)	0.000
19.	*Mystus* sp.	*Keḷutti*	Meat	Cold	Aphrodisiac	Bronchitis (1)	Kap. (1)	0.000
20.	*Nemipterus japonicas* (Bloch, 1791)	*Caṅkarā*	Meat	--	--	Chest pain (1)	Hea. (1)	0.000
21.	*Oreochromis mossambicus* (Peters, 1852)	*Jilēppi*	Meat	--	--	Bronchitis (1), Joint pain (1), Fatigue (1), Lactogogue (1), Chest pain (1)	Kap. (1), Vad. (1), Fat. (1), Gyn. (1), Hea. (1)	0.000
22.	*Pampus argenteus* (Euphrasen, 1788)	*Vavāl*	Meat	--	--	Cough (2), Lactogogue (1)	Kap. (2), Gyn. (1)	0.500
23.	*Parastromateus niger* (Bloch, 1795)	*Karuppu vavāl*	Meat	Hot	Aprhrodisiac and lactogogue	Wheezing (3), To increase memory (1)	Kap. (3), Psy. (1)	0.666
24.	*Parathelphusa hydrodromus* (Bloch, 1795)	*Cēṟṟu naṇṭu*	Meat	Hot	Stimulant, Febrifuge, To treat bronchitis, rheumatism and indigestion	Fever (3)	Fev. (3)	1.000
25.	*Parupeneus indicus* (Shaw, 1803)	*Nakarai/ mussara*	Meat	--	--	Coolant (1)	Coo. (1)	0.000
26.	*Portunus sanguinolentus* (Herbst, 1783)	*Kaṭal naṇṭu*	Meat	Hot	--	Bronchitis (3), Fever (2)	Kap. (3), Fev. (2)	0.750
27.	*Rachycentron canadus* (Linnaeus, 1766)	*Ney mīṉ*	Meat	--	--	Good for pregnant women (1), Lactogogue (1)	Gyn. (2)	1.000
28.	*Rastrelliger kanagurta* (Cuvier, 1816)	*Kāṉaṅkattai*	Meat	--	--	Bronchitis (3)	Kap. (3)	1.000
29.	*Sardinella longiceps* Valenciennes, 1847	*Peichālai*	Meat	--	--	Anabolic (4), Fatigue (1)	Anb. (4), Fat. (1)	0.750
30.	*Scoliodon laticaudus* Muller & Henle, 1838	*Piḷḷaiccuṟā*	Meat	Hot	Appitizer, lactogogue, To treat rheumatism and bronchitis	Lactogogue (7)	Gyn. (7)	1.000
31.	*Scomberomorus guttatus* (Bloch & Schneider, 1801)	*Vañciram*	Meat	--	--	Obesity (1), Fatigue (1), Chest pain (1), Rheumatalgia (1)	Obe. (1), Fat. (1), Hea. (1), Vad. (1)	0.000
32.	*Sphyraena jello* Cuvier in Cuvier & Valenciennes, 1829	*Ūḻi*	Meat	--	--	Lactogogue (1), Anabolic (2), Coolant (1)	Anb. (2), Gyn. (1), Coo. (1)	0.333
33.	*Stolephorus indicus* (van Hasselt, 1823)	*Nettili*	Meat	--	--	Cough (1), Wheezing (2), Lactogogue (2)	Kap. (3), Gyn. (2)	0.750
34.	*Stolephorus* spp.	--	Meat	--	--	Oligospermia (2)	Mal. (2)	1.000
35.	*Thryssa malabarica* (Bloch, 1795)	*Poruvā*	Meat	--	--	Arthritis (1)	Vad. (1)	0.000
36.	*Trichiurus lepturus* Linnaeus, 1758 *Eupleurogrammus muticus* (Gray, 1831)	*Ōlai vāḷai*	Meat	Hot	Appitizer, To treat bronchitis and rheumatism	Giddiness (1), Bronchitis (2), Joint pain (1)	Kap. (2), Hyp. (1), Vad. (1)	0.333
37.	*Upeneus sulphureus* Cuvier, 1829	*Navarai*	Meat	--	--	Joint pain (2)	Vad. (2)	1.000
38.	*Uroteuthis duvauceli* (d’Orbigny, 1835)	*Ūci kaṉavā*	Meat	--	--	Anemia (2), Rheumatalgia (1), Bronchitis (1)	Blo. (2), Vad. (1), Kap. (1)	0.333

^a^ - Data taken from *siddha materia medica* [43, 44]; Values given with in the parentheses indicate the number of UR for the respective illness/illness category

### Consensus over the use of plant-based medicinal foods

The illness categories viz., gastrointestinal ailments, hemorrhoids, and neural ailments were considered as illness categories with high consensus since they had higher FIC values than mean plus average FIC value. Sixteen illness categories such as *kapha* ailments, weakness, urinary, and ailments had average FIC values and were considered as illness categories with average consensus. Eight illness categories such as bone fractures, fatigue, fever, headache, hypotension, hypothyroidism, jaundice, and obesity were considered as illness categories with low consensus (Table 5).

**Table 5:** FIC values for illness categories treated with plant-based medicinal foods by the non-institutionally trained *siddha* practitioners in Tiruvallur district

Illness categories	Abbreviations used	*N*_UR_	% UR	*N*_*t*_	% *T*	*N*_*C*_	*N*_VC_	*F*_ic_
Analgesics	Ana.	18	3.94	14	13.46	14	3	0.235
Anabolic	Anb.	–	–	–	–	–	–	–
Blood ailments	Blo.	29	6.35	18	17.30	18	8	0.379
Bone fracture	Bon.	2	0.43	2	1.92	2	0	0.000
Coolants	Coo.	18	3.94	14	13.46	14	3	0.235
Dermatological ailments	Der.	5	1.09	4	3.84	4	1	0.250
Diabetes	Dia.	35	7.67	24	23.07	24	6	0.323
Eye ailments	Eye.	9	1.97	8	7.69	8	1	0.125
Fatigue	Fat.	14	3.07	14	13.46	14	0	0.000
Fever	Fev.	2	0.43	2	1.92	2	0	0.000
Gastrointestinal ailments	Gas.	90	19.73	36	34.61	36	26	0.606
Gynecological ailments	Gyn.	20	4.38	13	12.50	13	4	0.368
Headache	Hed.	1	0.21	1	0.96	1	0	0.000
Heart ailments	Hea.	21	4.60	15	14.42	15	4	0.300
Hemorrhoids	Hem.	20	4.38	8	7.69	8	6	0.613
Hypertension	Hpt.	13	2.85	10	9.61	10	3	0.250
Hypotension	Hpo.	8	1.74	8	7.69	8	0	0.000
Hypothyroidism	Thy.	3	0.65	3	2.88	3	0	0.000
Jaundice	Jau.	2	0.43	2	1.92	2	0	0.000
Kapha ailments	Kap.	32	7.01	17	16.34	17	10	0.483
Liver ailments	Liv.	4	0.87	3	2.88	3	1	0.333
Male reproductive ailments	Mal.	28	6.14	21	20.19	21	6	0.259
Neural ailments	Neu.	2	0.43	1	0.96	1	1	1.000
Obesity	Obe.	8	1.74	8	7.69	8	0	0.000
Psychological ailments	Psy.	8	1.74	7	6.73	7	1	0.142
Urinary ailments	Uri.	18	3.94	11	10.57	11	5	0.411
Vadha ailments	Vad.	11	2.41	9	8.65	9	2	0.200
Weakness	Wea.	35	7.67	19	6.50	19	15	0.470
Analgesics	Ana.	4	3.076	4	10.526	4	1	0.000
Anabolic	Anb.	11	8.461	5	13.157	5	2	0.600
Blood ailments	Blo.	4	3.076	2	5.263	2	2	0.666
Bone fracture	Bon.	2	1.538	1	2.613	1	1	1.000
Coolants	Coo.	6	4.615	5	13.157	5	1	0.200
Dermatological ailments	Der.	–	–	–	–	–	–	–
Diabetes	Dia.	–	–	–	–	–	–	–
Eye ailments	Eye.	3	2.307	3	7.894	3	0	0.000
Fatigue	Fat.	7	5.384	6	15.789	6	1	0.375
Fever	Fev.	6	4.615	3	7.894	3	2	0.166
Gastrointestinal ailments	Gas.	2	0.769	2	2.613	2	0	0.000
Gynecological ailments	Gyn.	19	14.615	10	26.315	10	4	0.500
Headache	Hed.	–	–	–	–	–	–	–
Heart ailments	Hea.	7	5.384	7	18.421	7	0	0.000
Hemorrhoids	Hem.	–	–	–	–	–	–	–
Hypertension	Hpt.	1	1.538	1	5.263	1	0	0.000
Hypotension	Hpo.	–	–	–	–	–	–	–
Hypothyroidism	Thy.	1	1.538	1	5.263	1	0	0.000
Jaundice	Jau.	–	–	–	–	–	–	–
Kapha ailments	Kap.	30	23.076	16	44.736	16	9	0.482
Liver ailments	Liv.	–	–	–	–	–	–	–
Male reproductive ailments	Mal.	11	6.923	8	21.052	8	3	0.714
Neural ailments	Neu.	–	–	–	–	–	–	–
Obesity	Obe.	2	1.538	2	2.613	2	0	0.000
Psychological ailments	Psy.	2	1.538	2	2.613	2	0	0.000
Urinary ailments	Uri.	3	2.564	2	2.613	2	1	0.500
Vadha ailments	Vad.	11	8.461	8	23.684	9	2	0.222
Weakness	Wea.	–	–	–	–	–	–	–

*N*_*UR*_ number of use reports, *% UR* % of use reports, *N*_*t*_ number of taxa, *% T* % of taxa, *N*_*c*_ number of claims, *N*_*vc*_ number of valid claims, *–* no UR and *F*_ic_ value was not calculated

### Consensus over the use of animal-based medicinal foods

Among the illness categories treated with animal-based medicinal foods, *kapha* ailments had gained high percentage (23.07%) of UR followed by gynecological ailments (14.61%). Nineteen illness categories were reported to be treated with animal-based medicinal foods. Analysis of the consensus showed that the categories viz., bone fractures, male reproductive ailments, blood ailments, and anabolic had high FIC values and were considered as illness categories with high consensus. Seven illness categories such as gynecological, urinary ailments, *kapha* ailments, fatigue, *vadha* ailments, coolants, and fever were grouped as illness categories with average consensus (Table 5).

### Illness categories treated with plant-based medicinal foods having high consensus

These two illness categories had high FIC scores among the illness categories treated with plant-based medicinal foods. Gastrointestinal ailment is the majorly cited illness category that represented 19.73% of total UR. In this category, 36 plant taxa were used to prepare the medicinal formulations. Among them, 26 taxa had a minimum of two UR for treating gastrointestinal ailments. The taxa such as fruits of *Citrus medica* (citron), leaves, unripe fruits of *Coccinia grandis* (ivy gourd), and flowers of *Musa paradisiaca* (plantain) had high number of UR.

In the case of hemorrhoids, eight plant taxa were used; among them, six taxa had a minimum of two UR. In this category, *Allium cepa* (onion), *Abutilon indicum*, *Amorphophallus paeoniifolius* (elephant foot yam), and plantain had high number of UR and IAR values. In the case of neural ailments, *Solanum americanum* had high number of UR and was reported to strengthen the nerves (Table 6).

**Table 6:** List of important plant and animal medicinal food taxa cited by the non-institutionally trained *Siddha* practitioners of Tiruvallur district, Tamil Nadu, to treat various ailments

Illness categories	Plants	Animals
Analgesics	*Cardiospermum halicacabum* (0.750), *Moringa oleifera* (0.470), *Solanum americanum* (0.409)	–
Anabolic	–	*Sardinella longiceps* (1.000), *Fenneropenaeus indicus* (0.500), *Sphyraena jello* (0.333)
Blood ailments	*Moringa oleifera* (0.470), *Beta vulgaris* (0.333), *Eclipta prostrata* (0.400), *Eleusine coracana* (0.333), *Ficus racemosa* (0.428), *Phyllanthus emblica* (0.384), *Trigonella foenum-graecum* (0.333), *Vitis vinifera* (0.375)	*Gallus gallus domesticus* (0.500), *Uroteuthis duvauceli* (0.333)
Bone fracture	–	*Capra aegagrus hircus* (0.333)
Coolants	*Borassus flabellifer* (0.666), *Coccinia grandis* (0.692), *Cuminum cyminum* (0.428)	*Bos taurus* (0.500)
Dermatological ailments	*Solanum americanum* (0.409)	–
Diabetes	*Coccinia grandis* (0.692), *Limonia acidissima* (0.750), *Abelmoschus esculentus* (0.333), *Brassica oleracea* var. *gongylodes* (1.000), *Marsilea quadrifolia* (0.666), *Syzygium cumini* (1.000)	–
Eye ailments	*Phyllanthus emblica* (0.384)	–
Fatigue	–	*Gibelion catla* (0.333)
Fever	–	*Parathelphusa hydrodromus* (1.000), *Portunus sanguinolentus* (0.750)
Gastrointestinal ailments	*Citrus medica* (0.666), *Coccinia grandis* (0.692), *Musa paradisiaca* (0.800) *Punica granatum* (0.444), *Solanum americanum* (0.409), *Citrus limon* (0.400), *Murraya koenigii* (0.428), *Ziziphus jujuba* (0.750), *Cuminum cyminum* (0.428), *Ipomoea aquatica* (0.750), *Mangifera indica* (0.666), *Phyllanthus emblica* (0.384), *Psidium guajava* (0.666), *Sesbania grandiflora* (0.285), *Vitis vinifera* (0.375), *Allium sativum* (0.250), *Citrullus lanatus* (0.500), *Cocos nucifera* (0.333), *Cucumis sativus* (1.000), *Daucus carota* (0.333), *Digera muricata* (1.000), *Ficus racemosa* (0.428), *Momordica charantia* (0.500), *Portulaca quadrifida* (0.400), *Solanum lycopersicum* (0.200), *Tamarindus indica* (1.000)	–
Gynecological ailments	*Aloe vera* (0.428), *Musa paradisiaca* (0.800), *Moringa oleifera* (0.470), *Vigna mungo* (0.444)	*Scoliodon laticaudus* (1.000), *Gibelion catla* (0.333), *Rachycentron canadus* (1.000), *Stolephorus indicus* (0.750)
Headache	–	–
Heart ailments	*Mangifera indica* (0.666), *Citrus medica* (0.666), *Hibiscus rosa-sinensis* (0.500), *Phyllanthus emblica* (0.384)	–
Hemorrhoids	*Allium cepa* (0.666), *Abutilon indicum* (1.000), *Amorphophallus paeoniifolius* (0.666), *Cissus quadrangularis* (0.400), *Musa paradisiaca* (0.800), *Acalypha indica* (0.333)	–
Hypertension	*Citrus medica* (0.666), *Moringa oleifera* (0.470), *Oxalis corniculata* (0.500)	–
Hypotension	–	–
Hypothyroidism	–	–
Jaundice	–	–
Kapha ailments	*Mukia maderaspatana* (1.000), *Solanum americanum* (0.409), *Cardiospermum halicacabum* (0.750), *Cleome gynandra* (0.250), *Leucas aspera* (0.250), *Moringa oleifera* (0.470), *Plectranthus amboinicus* (1.000), *Solanum torvum* (0.166), *Solanum trilobatum* (0.500)	*Himantura uarnak* (0.500), *Parastromateus niger* (0.666), *Rastrelliger kanagurta* (1.000), *Stolephorus indicus* (0.750), *Euthynnus affinis* (0.500), *Pampus argenteus* (0.500), *Trichiurus lepturus Eupleurogrammus muticus* (0.333)
Liver ailments	*Eclipta prostrata* (0.400)	–
Male reproductive ailments	*Solanum trilobatum* (0.500), *Allium cepa* (0.666), *Ficus racemosa* (0.428), *Ipomoea aquatica* (0.750), *Mangifera indica* (0.666), *Tribulus terrestris* (0.500)	*Capra aegagrus hircus* (0.500), *Katelysia opima* (0.500), *Stolephorus* spp. (1.000)
Neural ailments	*Solanum americanum* (0.409)	–
Obesity	–	–
Psychological ailments	*Centella asiatica* (0.500)	–
Urinary ailments	*Boerhavia diffusa* (1.000), *Lagenaria siceraria* (0.500), *Marsilea quadrifolia* (0.666), *Portulaca quadrifida* (0.400), *Tribulus terrestris* (0.500)	*Eleutheronema tetradactylum* (0.250)
Vadha ailments	*Citrus limon* (0.400), *Vigna mungo* (0.444)	*Lates calcarifer* (1.000), *Upeneus sulphureus* (1.0)
Weakness	*Vigna mungo* (0.444), *Amaranthus viridis* (0.333), *Anacardium occidentale* (0.500), *Arachis hypogaea* (0.500), *Cicer arietinum* (0.333), *Echinochloa frumentacea* (0.500), *Eleusine coracana* (0.333), *Oryza sativa* (0.250), *Panicum sumatrense* (0.500), *Paspalum scrobiculatum* (0.500), *Pennisetum glaucum* (0.333), Prunus dulcis (0.500), *Setaria italica* (0.500), *Sorghum bicolor* (0.500), *Zea mays* (0.500)	–

The values mentioned within the parentheses indicate the IAR values. Taxa are arranged in descending order of UR

### Illness categories treated with plant-based medicinal foods having average consensus

*Kapha* (pulmonary and respiratory) ailments gained 7.01% of total UR and 17 taxa. In this category, *Mukia maderaspatana* had high IAR score and UR. In the case of general weakness, the flour of *Vigna mungo* seeds (black gram) had high number of UR. The use of *Boerhavia diffusa* leaves had high UR to treat urinary ailments. The leaves of *Moringa oleifera* scored high UR under the category of blood ailments for the treatment of anemia. The gel of *Aloe vera* had high UR under the category of gynecological ailments and given to treat general ailments of uterus, dysmenorrhea, and metrorrhagia. In this category, the flowers and tender fruits of plantain had a high IAR score.

Cooked leaves of *Eclipta prostrata* were given to treat the liver ailments, and it had high number of UR in this category. In the case of diabetes, the plants such as stems of *Brassica oleracea* var. *gongylodes* (kohlrabi) and the fruits of *Syzygium cumini* had high IAR score. In this category, the leaves and fruits of ivy gourd had high number of UR. The fruits such as mango and citron had high number of UR and IAR score under the category of heart ailments. In the case of male reproductive ailments, the leaves of *Ipomoea aquatica* (water spinach) and onion had high IAR score; the use of flowers of *Solanum trilobatum* had high UR. The use of citron had high UR and IAR for the treatment of hypertension. Other important plants under this group were *Solanum americanum* (dermatological ailments), *Cardiospermum halicacabum* (analgesics), *Borassus flabellifer* (coolants), *Citrus limon* (*vadha* ailments), *Centella asiatica* (psychological ailments), and *Phyllanthus emblica* (eye ailments) (Table 6).

### Illness categories treated with animal-based medicinal foods having high consensus

The hoofs of *Capra aegagrus hircus* (goat) had been given to treat bone fractures. The use of milk and testes of goat, and the meat of *Katelysia opima* to treat male reproductive ailments had high UR; and the use of *Stolephorus* meat had high IAR score. The use of *Gallus gallus domesticus* eggs (chicken) and *Uroteuthis duvauceli* (Indian squid) had high UR under the category of blood ailments and were used to treat anemia. *Sardinella longiceps* (Indian oil sardine) and *Fenneropenaeus indicus* (Indian prawn) had high UR under the anabolics (Table 6).

### Illness categories treated with animal-based medicinal foods having average consensus

In the case of gynecological ailments, *Scoliodon laticaudus* (spade nose shark) had high UR and IAR value; it was reported to increase lactation. In the case of urinary ailments, *Eleutheronema tetradactylum* (fourfinger threadfin) had high UR and was reported to treat urolithiasis. In the case of *kapha* ailments, *Himantura uarnak* (reticulate whipray) had high number of UR; *Rastrelliger kanagurta* (Indian mackerel) and *Stolephorus indicus* (Indian anchovy) had high IAR values. In the case of fever, *Parathelphusa hydrodromus* and *Portunus sanguinolentus* (blood spotted swimming crab) had high UR and IAR scores. Other important animals under this category were *Gibelion catla* (catla) to treat fatigue, *Lates calcarifer* (barramundi) and *Upeneus sulphureus* to treat *vadha* ailments, and the buttermilk of *Bos taurus* (cow) as coolant (Table 6).

### Relationship between the humoral properties and illnesses

In the case of plant-based medicinal foods, the RFC for plants with cold humor was comparatively high (64.08%) compared to the plants with cold humor. In animal-based medications, no such variation was found. The illness categories such as analgesics, hemorrhoids, and *kapha* ailments had comparatively high RFC for plants with hot humor. In the case of animal-based foods, the categories such as fever, gynecological ailments, and *kapha* ailments have high RFC for plants with hot humor (Table 7).

**Table 7:** Frequency of URs for illness categories treated with plant and animal medicinal food taxa on the basis of humors

Illness categories	Plant food taxa			Animal food taxa		
	Hot	Cold	Unspecified	Hot	Cold	Unspecified
Analgesics	72.22	27.78	–	25	25	50
Anabolic	–	–	–	9.09	–	90.90
Blood ailments	24.13	68.97	6.90	–	50	50
Bone fracture	50	50	–	–	100	–
Coolants	11.11	88.89	–	–	33.33	66.66
Dermatological ailments	20	80	–	–	–	–
Diabetes	22.86	71.42	5.72	–	–	–
Eye ailments	33.33	66.67	–	33.33	33.33	33.33
Fatigue	14.28	78.58	7.14	–	57.14	42.85
Fever	50	50	–	83.33	–	16.66
Gastrointestinal ailments	35.56	62.22	2.22	–	100	–
Gynecological ailments	40	60	–	42.10	15.78	42.10
Headache	0	100	–	–	–	–
Heart ailments	52.35	42.65	5.00	0	0	100
Hemorrhoids	60	40	–	–	–	–
Hypertension	0	92.30	7.70	0	0	100
Hypotension	25	50	25	–	–	–
Hypothyroidism	33.33	66.67	–	100	0	0
Jaundice	0	100	–	–	–	–
Kapha ailments	59.37	40.63	–	36.66	6.66	56.66
Liver ailments	50	50	–	–	–	–
Male reproductive ailments	39.28	50	10.72	0	36.36	63.63
Neural ailments	50	50	–	–	–	–
Obesity	50	50	–	0	0	100
Psychological ailments	25	75	–	50	50	0
Urinary ailments	16.66	83.33	–	0	33.33	66.66
Vadha ailments	36.37	63.63	–	9.09	9.09	81.81
Weakness	22.85	71.42	5.73	–	–	–

*–* No UR and frequency was not calculated

### CFSI scores of the medicinal foods

List of plant and animal taxa having top ten CFSI scores are given in Table 8, and the CFSI scores for all taxa are given in Additional file 1: Table S2. It showed that the average CFSI score of the plant taxa was higher than that of the animal taxa.

**Table 8:** List of plant and animal taxa which got top ten CFSI score

Name of the taxa	CFSI
Plants	
*Solanum americanum*	110.28
*Murraya koenigii*	97.20
*Moringa oleifera*	95.64
*Cuminum cyminum*	63.84
*Musa paradisiaca*	55.68
*Coccinia grandis*	45.67
*Phyllanthus emblica*	45.36
*Solanum lycopersicum*	40.50
*Vitis vinifera*	40.24
*Punica granatum*	39.96
Animals	
*Portunus sanguinolentus*	33.75
*Sardinella longiceps*	29.25
*Fenneropenaeus indicus*	27.00
*Stolephorus indicus*	22.50
*Bos taurus*	19.50
*Rastrelliger kanagurta*	18.22
*Sphyraena jello*	16.20
*Scoliodon laticaudus*	15.75
*Himantura uarnak*	13.50
*Parastromateus niger*	13.50

## Discussion

In Indian systems of traditional medicine, diet recommendation is an integral and important part of the therapy; it is considered as an ally for strengthening the drug efficacy [46]. However, this knowledge is poorly documented and under-utilized. Our previous studies in other districts of Tamil Nadu also indicated that non-institutional training of *siddha* system is a male dominant domain [37], and cultural reasons play a vital role on low women’s participation. Such unevenness was also recorded in some ethnobiological studies in other geographical parts [47]. Traditional medicine was often perceived as the healthcare option only for the poor and marginalized communities; a recent work in Nepal indicated that these practices prevailed both in rural and semi-urban areas, and it showed positive correlation with household income and traditional medicine use [48]. The data of this study also indirectly substantiated the previous work by showing relatively high percentage of traditional healers in urban and semi-urban areas.

Sampling sufficiency and the representativeness of the samples collected are considered as major concerns of modern ethnobiology research [49], and various methods are employed to ascertain them. Application of species richness curve was one of the methods, and in this study, it was done by plotting Shannon-Wiener’s index in ordinate axis (*y*) and cumulative number of UR in abscissa axis (*x*). Reaching a clear asymptote of the curve was considered as an indicator for the sufficiency of sampling. Comparatively low Shannon-Wiener score for the animal-based foods indicated the lack of diversity in the animal foods than plant foods. The traditional medical literatures of *ayurveda* and *siddha* described about the health benefits of animal-based foods; however, the cultural and spiritual beliefs along with better understanding of the nutritional properties of plants caused a preference of vegetarianism in India [50]. Our previous study on ethnodietetics among non-institutionally trained *siddha* practitioners of Virudhunagar district had also yielded high number of UR for plant-based foods [12]. The same trend was reflected in this survey, and plant taxa got high UR and average IAR values. The CFSI scores for the plant taxa were comparatively higher than that of animal taxa. Our previous surveys in inland of Tamil Nadu showed low UR for fish taxa [12, 51]. In this survey, comparatively better availability of fish taxa caused more UR towards them.

Gastrointestinal ailments are one of the illnesses which got high number of UR in many ethnopharmacological explorations [52]. Food is directly related with various gastrointestinal illnesses, and the use of medicinal foods among subjects with functional gastrointestinal disorders was also high [53, 54]. The use of citron got a high number of UR in this study; it has also been used to treat gastrointestinal ailments in *ayurveda* and Chinese system of traditional medicine [55]. It has also been used for the treatment of the same in countries such as Nepal [56] and Pakistan [57]. A small clinical study with 37 subjects having recurrent aphthous stomatitis indicated that the application of citrus essential oil alleviated the pain in oral ulcers [58]. Some preliminary scientific experiments on *Citrus* fruits revealed the effectiveness on *Helicobacter pylori* [59]. From *Citrus* fruits, the compounds such as nobiletin [60], hesperidin, neohesperidin [61], β-myrcene [62], limonene, β-pinene [63], and 7,8-dimethoxycoumarin [64] were reported to have gastroprotective effects. Despite its traditional usage in many geographical areas and preclinical evidences, this claim lacks robust clinical data. Preliminary preclinical evidences supported the use of ivy gourd [65] and plantain for the treatment of gastric ulcer [66], but no clinical studies were available. The use of pomegranate was reported for the treatment of gastrointestinal ailments in Mexico [67] and Algeria [68]. *Solanum americanum* is one of the important plants of Tamil Nadu used to treat gastrointestinal ailments [69], and it is also used to treat gastrointestinal ailments in some other groups [70, 71]. Our previous ethnobotanical explorations had also documented the use of onion, *Abutilon indicum*, and elephant foot yam to treat hemorrhoids [37, 40, 72]; no scientific validation was reported.

Our previous surveys documented the use of *Mukia maderaspatana* in treating various pulmonary ailments [37, 40, 51, 72]. *Boerhavia diffusa* had been used to treat renal illnesses also in other geographic regions, and preclinical investigations showed its nephroprotective and antilithiasis effects [73, 74]. *Moringa oleifera* leaves are used as a supplement to treat anemia in other regions also [75], and according to a preclinical study, the dietary iron in *M. oleifera* is reported as superior to ferric citrate [76]. In *ayurveda* also, *Aloe vera* has been used to treat various gynecological ailments [77].

*Eclipta prostrata* is one of the important plants used in Indian as well as Chinese systems of traditional medicine for hepatoprotection [55]. Kohlrabi is an exotic taxon to India; it was not mentioned in the *siddha materia medica*, but it was prescribed by the informants of this study. Studies on such claims may yield some clues on knowledge transmission about the uses of exotic flora. Antidiabetic effect of *Brassica oleracea* was reported [78]; the antidiabetic effect of red kohlrabi was found to be superior to the green variety by a preclinical experiment [79]. A randomized, double blind trial with 63 type 2 diabetic subjects showed that the administration of broccoli (a variety of *Brassica oleracea*) powder at 10 g/day significantly lowered the insulin resistance [80]. Though *Syzygium cumini* was reported for diabetes by many previous studies, its antidiabetic efficacy was inconclusive [81]. The use of ivy gourd for the treatment of diabetes was also documented in Sri Lanka [82], Bangladesh [83], and Pakistan [84]. A small, double blinded phase I trial with 61 healthy volunteers indicated that consumption of 20 g of ivy gourd leaves significantly lowered the fasting and post-prandial glucose levels [85]. Pretreatment with mangiferin to isoproterenol induced myocardial infarcted rats prevented the alterations in mitochondrial energy metabolism and structural integrity of the heart tissues [86]. Cardioprotective effect of citron was recently reviewed [87]; no clinical reports were available to substantiate the cardioprotective effect of mango and citron supplementation. The use of *Citrus* fruits for the management of hypertension by Polish migrants in Argentina was already reported [88], and small double-blind, cross-over study with 12 stage I hypertensive patients indicated its usefulness [25]. Two small clinical studies showed the anxiolytic potential of *Centella asiatica* [89, 90].

The principle of using meat of an organ to treat the illnesses of the similar organ (*similia similibus curantur*) was reported in previous ethnopharmacological surveys [51, 91]. Previous studies in various geographical locations documented the use of testes and bones of goat to treat male reproductive [92] and bone [93] ailments, respectively. Geographic accessibility was one of the important factors that determined the popularity of zootherapy [94]; this study also represented that the accessibility of marine taxa caused more UR for them among the animal-based foods. Deb and Haque [95] documented the importance of fish taxa in the culture of people in coastal region; however, the ethnopharmacology of fish taxa in India still has to be documented thoroughly. Various medicinal properties of molluscs were recently reviewed [96]; this study documented the use of two molluscs viz., *Katelysia opima* and Indian squid for the treatment of impotence and anemia, respectively. Indian oil sardine has high ω-3 fatty acid content, high ω-3/ω-6 ratio, eicosapentaenoic acid, and docosahexaenoic acid [97], which may help to gain healthy weight.

The use of spade nose shark to improve lactation had got high UR and IAR under gynecological ailments; however, no scientific report is available to validate this claim. The study by Deb and Haque [95] documented the use of catla as lactogogue, *Anguilla bengalensis bengalensis* for the treatment of arthritis, and *Channa* spp., for oligospermia. They also documented reticulate whipray as lactogogue and for the treatment of dysentery; this survey documented its usefulness to treat wheezing and bronchitis.

## Conclusions

- This preliminary report quantitatively documented the food-medicine continuum among the non-institutionally trained *siddha* practitioners of Tiruvallur district. Collectivistic cultures, influence of traditional norms, and medicinal beliefs caused Indian dietary habits to be very unique; this provides ample scope for further research to anthropologists and ethnobiologists. Deeper studies on different dietary cultures of India may help derive better interpretations on food-medicine continuum.
- This study identified some important plant-based medicinal claims such as citron, pomegranate and *Solanum americanum* (gastrointestinal ailments), *Abutilon indicum*, onions and elephant foot yam (hemorrhoids), *Boerhavia diffusa* (urinary ailments), *Moringa oleifera* (anemia), *Aloe vera* (gynecological ailments), *Eclipta prostrata* (liver ailments), ivy gourd (diabetes), citron (hypertension), and *Centella asiatica* (psychological ailments). More studies on these claims will help identify novel functional foods to add to the field of medical nutrition therapy, with traditional brand identity.
- This study also documented some important marine animal taxa such as spade nose shark (lactogogue), reticulate whipray (wheezing and bronchitis), *Katelysia opima* (impotence), Indian squid (anemia), and Indian oil sardine (anabolic) for the treatment of various illnesses. Generally, ethnopharmacological validations on ethnozoological claims are very meager and Indian marine resources are still under-utilized. Scientific studies on these claims may yield some novel and affordable functional foods.
- Documentation of ethnopharmacological knowledge of marine resources is comparatively low in India. Indian coastal line spreads over 7516 km, and robust studies on the documentation of the traditional knowledge on marine resources will yield a good database for various stakeholders and policy makers.
- Among zootherapy, the use of organs to treat illnesses of similar organs was documented in many instances. Future-specific studies will reveal the cultural and pharmacological importance of this claim.

## Additional file

Additional file 1: **Table S1.** List of medicinal foods prescribed by the non-institutionally trained *siddha* practitioners of Tiruvallur district of Tamil Nadu. The taxa given in bold emphasis are mentioned as the key taxa for the reported medicinal activity of the food by the informants. The values given within the parentheses indicate the number of the UR for the respective illness. **Table S2**. Cultural Food Significance Index of the plant and animal taxa cited by the non-institutionally trained *siddha* practitioners of Tiruvallur district of Tamil Nadu for preparing medicinal foods. Taxa having a minimum of two UR were taken for the analysis. **AI** Availability Index, **QI** Quotation Index, **UFI** Utilization Frequency Index, **PUI** Parts Used Index, **MFFI** Multi-Functional Food Use Index, **TASI** Taste Score Appreciation Index, **FMRI** Food-Medicinal Role Index, **CFSI** Cultural Food Significance Index (DOCX 74 kb).

## Abbreviations

AI: Availability index
CFSI: Cultural Food Significance Index
FIC: Informant Consensus Factor
FMRI: Food Medicinal Role Index
FUI: Frequency of use index
IAR: Index of Agreement on Remedies
MFFI: Multifunctional food use index
*n*_*a*_: Number of illness categories treated with that taxon
*N*_*t*_: Number of taxa
*N*_ur_: Number of UR
*n*_ur_: Number of UR registered for taxa
PUI: Part used index
QI: Quotation index
RFC: Relative Frequency of Citation
TSAI: Taste Score Appreciation Index
UR: Use report
