# Supplementary material for: A study on food-medicine continuum among the non-institutionally trained siddha practitioners of Tiruvallur district, Tamil Nadu, India
Source: J Ethnobiol Ethnomed. 2018 Jun 28;14:45. doi: 10.1186/s13002-018-0240-9 (PMC6025710; doi:10.1186/s13002-018-0240-9)
Supplement: Supplementary file 1 — Table S1. List of medicinal foods prescribed by the non-institutionally trained siddha practitioners of Tiruvallur district of Tamil Nadu. The taxa given in bold emphasis are mentioned as the key taxa for the reported medicinal activity of the food by the informants. The values given within the parentheses indicate the number of the UR for the respective illness. Table S2. Cultural Food Significance Index of the plant and animal taxa cited by the non-institutionally trained siddha practitioners of Tiruvallur district of Tamil Nadu for preparing medicinal foods. Taxa having a minimum of two UR were taken for the analysis. AI Availability Index, QI Quotation Index, UFI Utilization Frequency Index, PUI Parts Used Index, MFFI Multi-Functional Food Use Index, TASI Taste Score Appreciation Index, FMRI Food-Medicinal Role Index, CFSI Cultural Food Significance Index (DOCX 74 kb). [file 13002_2018_240_MOESM1_ESM.docx]

**Table S1**: List of medicinal foods prescribed by the non-institutionally trained *siddha* practitioners of Tiruvallur district of Tamil Nadu

| **S.No** | **Ingredients** | **Mode of preparation** | **Dose & Duration** | **Illnesses** |
| --- | --- | --- | --- | --- |
|  | **Seeds of** ***Macrotyloma uniflorum*** (25 g.), **Fruits of** ***Garcinia gummi-gutta*** (25 g.) , Leaves of *Murraya koenigii* (q.s.), Bulbils of *Allium sativum*, Seeds of *Piper nigrum* (q.s.), Salt (q.s.) | The seeds of *M. uniflorum* are made into decoction and the pulps of *G. gummi-gutta* fruits are smashed in the decoction and filtered. The decoction is added with crushed other ingredients, boiled and made into a soup (*rasam*) | Consumed with food, once a day | Obesity (1) |
|  | **Petals of *Hibiscus rosa-sinensis*** Honey (q.s.) | The petals are soaked in honey, kept in sunlight for a week and stored | Approximately 5 g., Once in the morning for 45 days | Impotence (1) |
|  | **Petals of *Hibiscus rosa-sinensis*** Fruit juice of *Citrus limon* (q.s.), Honey (q.s.) | Cleaned petals are soaked in lemon juice overnight, the juice is filtered, added with equal quantity of honey and made into syrup | 15 – 20 mL., with milk twice a day for 45 days | Heart diseases (2) |
|  | **Petals of *Senna auriculata*.** (500 g) , Cow’s milk (l liter), Palm sugar (1 kg) | The petals are cleaned, boiled in milk, added with palm sugar and made into a jam | 5 g., twice a day for three months | Impotence (1), Lumbo-sacral pain (1) |
|  | **Fruits of *Lagenaria siceraria*** (100 g), Seeds of *Piper nigrum* (q.s.), Salt (q.s.) | The pulp from the tender fruits are made into soup | Consumed once a day in the morning, twice a week | Obesity (1) |
|  | **Peals of *Nelumbo nucifera*** | The petals are shade dried and powdered. One teaspoonful of powder is made into decoction and consumed with milk and honey | Once a day in the morning for 45 days | Heart diseases (1) |
|  | **Leaves of *Murraya koenigii*** (20 g), **Bulbils of *Allium sativum*** (2 g), Seeds of *Piper nigrum* (q.s.), Salt (q.s.) | The leaves are fried in clarified butter, added with other ingredients and are made into a sauce (*Chutney*) | Consumed with food | Indigestion (1), Dysmenorrhea (1), Diabetes (1) |
|  | **Gel of *Aloe vera*** Buttermilk (q.s.) | The leaves are skinned, the gel is washed repeatedly with water for seven times, crushed, the juice is added equal amount of buttermilk | 50 mL., once in the morning for 48 days before food | Cholelithiasis (1), Urolithiasis (1), Liver diseases (1), Dysmenorrhea (1), Metrorrhagia (1) |
|  | **Leaves of *Cardiospermum halicacabum*** (10 g), **Leaves of *Solanum trilobatum*** (10 g), **Leaves of *Moringa oleifera*** (10 g), **Leaves of *Leucas aspera*** (10 g), Bulbils of *Allium sativum* (2 g), Dried fruits of *Capsicum annuum* (q.s.), Seeds of *Piper nigrum* (q.s.), Seeds of *Cuminum cyminum* (q.s.), Fruits of *Tamarindus indica* (q.s.) | The ingredients are crushed, added with the juice of *T. indica,* boiled and made as a soup | Consumed with food, once a day for three days | Rhinitis (1), Cough (1), Somatalgia (1) |
|  | **Boiled, skin removed rhizome of *Amorphophallus paeoniifolius*** (50 kg), Fruits of *Tamarindus indica* (q.s.), Bulbils of *Allium sativum* (q.s.), Bulbils of *Allium cepa* (q.s.), Seeds of *Cuminum cyminum* (q.s.), Seeds of *Piper nigrum* (q.s.), Salt (q.s.) | Garlic pearls, onion, cumin seeds and peppercorns are roased in clarified butter, added with boiled rhizomes of  *A. paeoniifolius,* water and salt and made into sauce | Once a day with lunch for 30 days | Hemorrhoids (3), Hypertension (1) |
|  | **Gel of *Aloe vera***  (2 Kg), Sugar from *Borassus flabellifer* (1.5 kg), Clarified butter (250 g) | The gel of *A. vera* was washed in water for seven times, added, fried in clarified butter, added with palm sugar and made into jam | 5 g., twice a day with milk, till cure | Dysmenorrhea (1), Uterus disorders (1), Gastric ulcers (1) |
|  | **Bulbils of *Allium sativum*** (10 g), **Seeds of *Foeniculum vulgare*** (1 g), **Seeds of *Trachyspermum ammi*** (1 g), Cow’s milk (50 mL) | The first three ingredients are crushed, boiled in the milk and consumed with honey | Once in the morning before food, for 40 days | Heart disease (1) |
|  | **Seeds of *Sesamum indicum*** (100 g) | The seeds are slightly roasted and consumed with milk | Approximately 10 g., twice a day | Impotence (1) |
|  | **Leaves of *Amaranthus viridis*** (100 g), Bulbils of *Allium sativum* (q.s.), Fruits of *Solanum lycopersicum* (q.s.), Blubs of *Allium cepa* (q.s.), Seeds of *Cuminum cyminum* (q.s.), Seeds of *Piper nigrum* (q.s.), Salt (q.s.), Clarified butter (q.s.) | The leaves are boiled and smashed. The other ingredients are crushed, roasted in clarified butter and added with the leaves to make as gravy | Once a day, twice a week | Impotence (1), Anemia (1), General weakness (2) |
|  | **Seeds of *Eleusine coracana*** (250 g), **Seeds of *Pennisetum glaucum*** (250 g), **Seeds of *Paspalum scrobiculatum*** (100 g), **Seeds of *Panicum sumatrense*** (100 g), **Seeds of *Setaria italica*** (100 g), **Seeds of *Echinochloa frumentacea*** (100 g), **Seeds of *Sorghum bicolor*** (100 g), **Seeds of *Zea mays*** (50 g), **Seeds of *Arachis hypogaea*** (100 g), **Seeds of *Anacardium occidentale*** (100 g), **Seeds of *Prunus dulcis*** (100 g), **Roasted seeds of *Cicer arietinum*** (10 g), **Hand pounded rice of *Oryza sativa*** (100 g), Dried fruits of *Elettaria cardamomum* (10 g) | The ingredients are powdered and made into porridge with milk | As breakfast when needed | General weakness (2), Fatigue (1) |
|  | **Dried rhizomes of *Zingiber officinale* Roscoe** (25 g), **Seeds of *Piper nigrum*** (10 g), **Seeds of *Piper longum*** (10 g), **Seeds of *Cuminum cyminum*** (10 g), **Seeds of *Nigella sativa*** (10 g), **Seeds of *Trachyspermum ammi*** (10 g), **Rock salt** (5 g), **Resin of *Ferula assa-foetida*** (5 g) | The ingredients are powdered and about 2 g., of the powder is made into decoction | Once or twice a day, as needed | Somatalgia (1) |
|  | **Stem of *Cissus quadrangularis*** (100 g), Fruits of *Tamarindus indica* (10 g), Leaves of *Murraya koenigii* (10 g), Bulbils of *Allium sativum* (15 g), Dehusked seeds of *Cajanus cajan* (25 g), Clarified butter (10 g), Dry fruits of *Capsicum annuum* (q.s.), Salt (q.s.) | The internodes and the skin of *C. quadrangularis* are removed and fried in clarified butter. The skin of the ginger is also removed and fried in clarified butter. Then the stems are ground with other ingredients as sauce (*chutney*) | Consumed once with lunch for 30 days | Obesity (1), Gastric ulcers (1), Bone fractures (1), Hemorrhoids (3) |
|  | **Bulbils of *Allium sativum*** (15 g) | The bulbils are roasted in clarified butter | Consumed with food, once a day for 5 days | Flatus (1) |
|  | **Tender leaves of *Erythrina variegata*** (25 g), *idli* batter (q.s.) | The leaves are chopped, added with *idli* batter and made into *dosa* | Once in the morning for three days | Bronchitis (1) |
|  | **Leaf juice of *Cocculus hirsutus*** (25 mL), Seeds of *Elettaria cardamomum* (0.5 g), Palm sugar (25 g) | The leaf juice is mixed with palm sugar, powdered cardamom seeds and a cup of water. This mixture is kept for an hour till it gets gel like consistency and then consumed | Once in the morning as required | Oligospermia, Body heat |
|  | **Fruits of *Ficus racemosa***, **Fruits of *Ficus religiosa***, **Fruits of *Ficus benghalensis*** (in equal quantities), Honey (q.s.) | The fruits are cut into pieces and pickled in honey | Approximately 10 g., with milk, once in night for three months | Oligospermia (1) |
|  | **Leaves of *Plectranthus amboinicus*** (q.s.), Flour of *Cicer arietinum* (q.s.), Dry fruits of *Capsicum annuum* (q.s.), Resin of *Ferula assa-foetida* (q.s.), Salt (q.s.) | Powdered chili, asafetida, salt and water are added with chickpea flour and made as batter; the leaves are dipped in the batter, fried in oil and eaten as a snack (*Bhaji*) | As needed | Rhinitis (1), Bronchitis (1) |
|  | **Dehusked seeds of *Vigna mungo*** (25 g), Clarified butter (q.s.), Honey (q.s.) | The seeds are ground as batter, mixed with salt and pepper, fried in clarified butter as *vada* (a fried snack), mixed with honey and consumed | Once a day, with breakfast | General weakness (3), Burning sensation (1) |
|  | **Flowers of *Musa paradisiaca*** (50 g), Seeds of *Piper nigrum* (q.s.), Salt (q.s.) | The flowers are cleaned, gynoecium and bracts are removed, slightly fried, added with pepper and salt and ground well as *chutney* | Once a day with lunch, for three days | Menstrual cramps (2), gastric ulcers (3), mouth ulcers (1) |
|  | **Tender fruits of *Musa paradisiaca*** (50 g), Buttermilk (q.s.) | The skin of *M. paradisiaca* fruits are removed, crushed with buttermilk, filtered and the juice is consumed | Once in the morning for three days | Menstrual cramps (1) |
|  | **Flowers of *Musa paradisiaca*** (50 g), Buttermilk (q.s.) | The flowers are cleaned, gynoecium and bracts are removed, crushed with buttermilk, filtered and the juice is consumed | Once in the morning for a week | Hyperacidity (1) |
|  | **Dried fruits *Solanum torvum*** (25 g), Fruits of *Tamarindus indica* (10 g), Bulbils of *Allium sativum* (15 g), Bulbils of *Allium cepa* (15 g), Dry fruits of *Capsicum annuum* (q.s.), Resin of *Ferula assa-foetida* (q.s.), Leaves of *Murraya koenigii* (q.s.), Powdered rhizomes of *Curcuma longa* (q.s.), Oil of *Sesamum indicum* (q.s.), Salt (q.s.) | The fruits of *S. torvum,* chopped onion, garlic, curry leaves, asafetida are fried in gingelly oil, the juice of tamarind is added, powdered chili and salt are added, boiled and made as a gravy | Given with food, once a day, twice a week | Hypotension (1), Heart diseases (1), Bronchitis (1) |
|  | **Dried fruits *Solanum torvum*** (10 g) | The fruits are fried in oil and given with food as side dish | Once with supper, twice a week | Anemia (1), Wheezing (1), Diarrhea (1), Diabetes (1) |
|  | **Leaves of *Coccinia grandis*** (25 g), Grated endosperm of *Cocos nucifera* (25 g), Oil of *Sesamum indicum* (q.s.), Seeds of *Piper nigrum* (q.s.), Salt (q.s.) | The leaves are cleaned, chopped into pieces, boiled, added with coconut, pepper and salt and slightly fried in sesame oil | Once a day with lunch, till cure | Gastric ulcers (2), Mouth ulcers(1), Oliguria (1), Diabetes(1), Burning sensation (1) |
|  | **Tender, unripe fruits of *Coccinia grandis*** (50 g), Seeds of *Piper nigrum* (q.s.), Salt (q.s.), Curd (q.s.) | The fruits are chopped, added with other ingredients and consumed as salad | Twice a day, till cure | Gastric ulcers (1), Mouth ulcers (1), Diabetes (2) |
|  | **Fruit Pulp of *Limonia acidissima*** (50 g), Jaggery from *Borassus flabellifer* (q.s.) | The pulp is mixed with palm jaggery and consumed | When available | Diabetes (3), Polydipsia (1), Hypertension (1) |
|  | **Unripe fruits of *Momordica charantia*** (50 g), Dry fruits of *Capsicum annuum* (q.s.), Salt (q.s.) | The fruits are chopped, mixed with powdered chili and salt and fried in oil | Consumed with food | Diabetes (1), Bloating (1), Intestinal worms (1) |
|  | **Ripe fruits of *Ficus racemosa*** | Consumed as raw | Twice a day | Anemia (1), Impotence (1), Constipation (1), Wheezing (1) |
|  | **Leaves of *Moringa oleifera*** (50 g), Grated endosperm of *Cocos nucifera* (25 g), Cotyledons of *Arachis hypogaea* (10 g), Dry fruits of *Capsicum annuum* (q.s.), Oil of *Arachis hypogaea* (q.s.), Salt (q.s.) | The leaves are cleaned, boiled in water, added with other ingredients and slightly fried in oil | Once a day with lunch, thrice a week | Anemia (2), General weakness (1), To increase vision (1) |
|  | **Petioles of *Moringa oleifera* Lam.** (50 g), Fruits of *Solanum lycopersicum* (50 g), Fruits of *Tamarindus indica* (15 g), Bulbils of *Allium sativum* (10 g), Bulbils of *Allium cepa* (10 g), Seeds of *Cuminum cyminum* (5 g), Seeds of *Piper nigrum* (2 g), Dry fruits of *Capsicum annuum* (q.s.), Resin of *Ferula assa-foetida* (q.s.), Salt (q.s.), Oil (q.s.) | The petioles are crushed with garlic, cumin seeds, pepper, chili and tomato. Onion and asafetida are fried in oil, tamarind juice and other ingredients are is added, boiled and made into a soup | Once a day, with food | Hypertension (2), Somatalgia (1) |
|  | **Leaves of *Moringa oleifera*** (50 g), Grated endosperm of *Cocos nucifera* (25 g), Oilcake of *Sesamum indicum* (25 g), Dry fruits of *Capsicum annuum* (q.s.), Oil of  *Sesamum indicum* (q.s.), Salt (q.s.) | The leaves are cleaned, boiled in water, added with other ingredients and slightly fried in oil | Once a day, twice a week | Diabetes (1) |
|  | **Rhizomes of *Beta vulgaris*** (50 g) | The juice is consumed | Once a day for a week | Anemia (1) |
|  | **Roasted seeds of *Cicer arietinum*** (100 g), Jaggery from *Borassus flabellifer* (100 g), Clarified butter (q.s.) | The ingredients are mixed and made into small balls | Eaten when needed | Menstrual cramps (1) |
|  | **Fruits of *Syzygium cumini*** | The fruits are consumed as raw | When available | Diabetes (2) |
|  | **Deseeded fruits of *Phyllanthus emblica***, Honey (in equal quantities) | The pulp of *P. emblica* is pickled in honey | Twice a day, with milk | Heart diseases (2), Anemia (1), Constipation (1) |
|  | **Leaves of *Moringa oleifera*** (10 g), **Leaves of *Murraya koenigii*** (10 g), Bulbils of *Allium sativum* (q.s.), Seeds of *Piper nigrum* (q.s.), Salt (q.s.) | The ingredients are crushed and made as a soup | Once in the evening | Anemia (1), Obesity (1), Anorexia (1) |
|  | **Juice from the rhizomes of *Daucus carota*** (100 mL), **Juice from the rhizomes of *Beta vulgaris*** (50 mL), **Juice from the endosperm of *Cocos nucifera*** (50 mL), **Juice from the fruits of *Vitis vinifera*** (50 mL), **Deseeded fruits of *Phoenix dactylifera*** (50 g), Cow’s milk (100 mL), Honey (100 mL) | The fruits of *P. dactylifera* are ground in milk, mixed with other ingredients and made into syrup. | 10 mL., with milk twice a day for three months | Impotence (1), Hypotension (1) |
|  | **Dehusked seeds of *Vigna mungo*** (50 g), Jaggery from the inflorescence of *Borassus flabellifer* (50 g), Oil of *Sesamum indicum* (50 mL), Cow’s milk (250 mL) | Dehusked black gram seeds are slightly fried and powdered. It is added with the other ingredients, boiled in small flame and made as a jam (*kali*) | Twice a day, for 20 days | Cervicalgia (1), Lumbalgia, Ovulation problems (1), As supplement after puberty (1) |
|  | **Leaves of *Centella asiatica*** (50 g), Bulbils of *Allium cepa* (20 g), Bulbils of *Allium sativum* (10 g), Seeds of *Piper nigrum* (q.s.), Salt (q.s.), Resin of *Ferula assa-foetida* (q.s.), Dried fruits of *Capsicum annuum* (q.s.), Dried rhizome powder of *Curcuma longa*  (q.s.), Oil of *Sesamum indicum* (q.s.), Salt (q.s.) | The leaves are cleaned, chopped into pieces, boiled in water and smashed. Garlic pearls and onion are chopped into pieces; other ingredients are powdered, fried in oil and added with smashed leaves of *C. asiatica* | Once a day for 15 days | Reduced memory (2), Hypothyrodism (1) |
|  | **Leaves of *Solanum americanum*** (50 g), Bulbils of *Allium cepa* (q.s), Bulbils of *Allium sativum* (q.s), Seeds of *Piper nigrum* (q.s), Seeds of *Cuminum cyminum* (q.s), Dried rhizome powder of *Curcuma longa* (q.s), Oil of *Sesamum indicum* (q.s), Salt (q.s) | The ingredients are made into soup | Once in the evening | Menstrual migraine (1) |
|  | **Leaves of *Moringa oleifera*** (50 g), Bulbils of *Allium cepa* (q.s), Bulbils of *Allium sativum* (q.s), Seeds of *Piper nigrum* (q.s), Seeds of *Cuminum cyminum* (q.s), Dried rhizome powder of *Curcuma longa* (q.s), Oil of *Sesamum indicum* (q.s), Salt (q.s) | The ingredients are made into soup | Once in the evening | Menstrual migraine (2) |
|  | **Leaves of *Alternanthera sessilis*** (50 g), Bulbils of *Allium cepa* (q.s), Bulbils of *Allium sativum* (q.s), Seeds of *Piper nigrum* (q.s), Seeds of *Cuminum cyminum* (q.s), Dried rhizome powder of *Curcuma longa* (q.s), Oil of *Sesamum indicum* (q.s), Salt (q.s) | The ingredients are made into soup | Once in the evening | Menstrual migraine (1) |
|  | **Leaves of *Solanum americanum*** (50 g), Leaves of *Coriandrum sativum* (25 g), Bulbils of *Allium sativum* (q.s.), Dried rhizomes of *Zingiber officinale* (q.s.), Seeds of *Piper nigrum* (q.s), Salt (q.s.) | The ingredients are fried slightly, ground into a paste (*chutney*) | Once a day, with lunch | To strengthen bones and nerves (1), Bronchitis (1), Somatalgia (1), Heart diseases (1), Wheezing (1) |
|  | **Leaves of *Mukia maderaspatana*** (50 g), Leaves of *Coriandrum sativum* (q.s.), Bulbils of *Allium sativum* (q.s.), Dried rhizome of *Zingiber officinale* (q.s.), Salt (q.s), Egg (1 No.) | The ingredients are chopped, slightly fried, mixed with egg and *dosa* batter and made into *dosa* | Once in the morning | Asthma (2), Bronchitis (2), Cough (1) |
|  | **Pulps of *Phyllanthus emblica*** (50 g), Bulbils of *Allium sativum* (15 g), Rhizomes of *Zingiber officinale* (15 g), Seeds of *Vigna mungo* (15 g), Dried fruits of *Capsicum annuum* (q.s.), Salt (q.s.) | The ingredients are slightly fried in oil, ground and made into a *chutney* | Once a day for 15 days | Asthma (1) |
|  | **Leaves of *Hedyotis puberula*.** (25 g), Seeds of *Piper nigrum* (q.s.), Salt (5 g) | The leaves and pepper seeds are crushed, mixed with *dosa* batter and prepared as a *dosa* | Twice a day for 15 days | Productive cough (1), Bronchitis (1), Sinusitis (1) |
|  | **Tender, unripe fruits of *Ficus racemosa*** (100 g), Dehusked seeds of *Vigna radiata* (50 g), Bulbils of *Allium sativum* (q.s.), Bulbils of *Allium cepa* (q.s.), Leaves of *Murraya koenigii* (q.s.), Seeds of *Piper nigrum* (q.s), Dried fruits of *Capsicum annuum* (q.s.), Clarified butter (q.s.), Salt (q.s.) | The fruits are cleaned and boiled with green gram. The other ingredients are chopped/powdered and fried in clarified butter. Both are mixed and smashed | Consumed once with lunch, for 40 days | Gastric ulcers (1), Hemorrhoids (1), Anemia (1) |
|  | **Leaves of *Hybanthus enneaspermus*** (100 g), Dehusked seeds of *Vigna radiata* (50 g), Bulbils of *Allium sativum* (q.s.), Bulbils of *Allium cepa* (q.s.), Leaves of *Murraya koenigii* (q.s.), Seeds of *Piper nigrum* (q.s), Dried fruits of *Capsicum annuum* (q.s.), Clarified butter (q.s.), Salt (q.s.) | The leaves are cleaned and boiled with green gram. The other ingredients are chopped/powdered and fried in clarified butter. Both are mixed and smashed | Consumed once with lunch, for 40 days | Oligospermia (1), Hypothyroidism (1) |
|  | **Seeds of *Trigonella foenum-graecum*** (100 g), **Seeds of *Sesamum indicum*** (100 g), **Dehusked seeds of *Vigna mungo*** (100 g), **Seeds of *Eleusine coracana*** (250 g), **Seeds of *Triticum aestivum*** (250 g), **Hand pounded rice (*Oryza sativa*)** (250 g), Seeds of *Papaver somniferum* (25 g), Seeds of *Foeniculum vulgare* (25 g), Seeds of *Cuminum cyminum* (10 g), Seeds of *Trachyspermum ammi* (25 g) | The ingredients are slightly fried and powdered. Required amount of this flour is made as porridge/*roti* | As breakfast | Diabetes (1) |
|  | **Leaves of *Sesbania grandiflora***  (50 g), Bulbils of *Allium sativum* (q.s.), Seeds of *Cuminum cyminum* (q.s.), Seeds of *Piper nigrum* (q.s.), Dried rhizome powder of *Curcuma longa* (q.s.), Salt (q.s.), | The ingredients are made into a soup | Once a day | Obesity (1), Gastric ulcers (3), Hemorrhoids (1) |
|  | **Juice of the rhizomes of *Daucus carota*** (25 mL), Juice of the rhizomes of *Beta vulgaris* (25 mL), Juice of the fruits of *Solanum lycopersicum* (25 mL), Sugar from *Borassus flabellifer* (q.s.) | The juices are mixed with required quantity of palm sugar and consumed | Once or twice, for two days | Bloating (1), Nausea (1) |
|  | **Fruits of *Phoenix dactylifera***, Honey (q.s.) | The fruits are chopped into pieces and pickled in honey | Once in the evening with milk | General weakness (1) |
|  | **Fruits juice of *Punica granatum*** (50 mL), Milk (q.s.), Honey (q.s.) | The ingredients are mixed well and consumed | Once a day | Bromhidrosis (1), Hyperacidity (1) Constipation (1), Gastric ulcers (1), Anorexia (2), Asthma (1), Rheumatalgia (1), To increase vision (1), Hypothyroidism (1) |
|  | **Fruit juice of *Citrus limon*** (from one fruit), Honey (q.s.), Salt (q.s.), Water (q.s.) | The ingredients are made into a juice | Once or twice | Indigestion (3), Constipation (1), Heart diseases (1), Acne (1), Diabetes (1), Arthritis (2), To increase vision (1), Hypothyroidism (1) |
|  | **Fruit juice of *Vitis vinifera*** (50 mL), Honey (q.s.) | The ingredients are mixed well and consumed | Once in the morning for 30 days | Anemia (2), Anorexia (2), Constipation (1), Wheezing (1), Rheumatalgia (1) |
|  | **Fruit juice of *Phyllanthus emblica*** (50 mL), Honey (q.s.) | The ingredients are mixed well and consumed | Once in the morning for 30 days | Anemia (1), Burning sensation (1), Diabetes (1), Anorexia (2), Rheumatalgia (1), Blurred vision (2), Hypothyroidism (1) |
|  | **Sugar from the inflorescence of *Borassus flabellifer*** (2 g), Milk of tender fruits of *Cocos nucifera* (q.s.) | Palm sugar is dissolved in tender coconut milk and consumed | Once or twice for a day, for seven days | Burning sensation (3), Jaundice (1) |
|  | **Seeds of *Gossypium*** (100 g), Dried fruits of *Elettaria cardamomum* (5 g), Sugar from *Borassus flabellifer* (q.s.) | The seeds are soaked overnight; ground well with cardamom seeds and the juice of is extracted. Sufficient quantity of palm sugar is added, filtered slightly boiled and consumed | Once daily | General weakness (1), Somatalgia (1) |
|  | **Tender fruits of *Coccinia grandis*** (50 g), Curd of  *Bos taurus* | The fruits are cleaned, chopped and mixed with curd | Once a day | Burning sensation (1) |
|  | **Bulbils of *Allium cepa*** (50 g), Clarified butter (q.s.) | Onion bulbils are chopped and slightly fried in clarified butter. It is mixed with rice and consumed | Once in the morning, for 90 days | Hemorrhoids (1), Bleeding through rectum (3), Oligospermia (2), Burning sensation(1) |
|  | **Unripe, tender fruits of *Lagenaria siceraria*** (100 g), Dehusked seeds of *Vigna radiata* (50 g), Bulbils of *Allium sativum* (q.s.), Bulbils of *Allium cepa* (q.s.), Leaves of *Murraya koenigii* (q.s.), Dried fruits of *Capsicum annuum* (q.s.), Oil (q.s.), Salt (q.s.) | The ingredients are made into a gravy | Once a day with food | Coolant (1), To strengthen memory (1), Swelling of the limbs (1), Dysuria (2), |
|  | **Sprouted seeds of *Trigonella foenum-graecum*** (50 g), Grated endosperm of *Cocos nucifera* (50 g), Dry fruits of *Capsicum annuum* (q.s.), Oil of *Arachis hypogaea* (q.s.), Salt (q.s.) | The sprouts of fenugreek with two cotyledonary leaves are cleaned, added with coconut, slightly fried in the oil, required amount of chili and salt are added and consumed | Once a day | Burning sensation (1), Anemia (1) |
|  | **Leaves of *Mentha arvensis*** (100 g), Fruits of *Tamarindus indica* (15 g), rhizome of *Zingiber officinale* (15 g), Dry fruits of *Capsicum annuum* (q.s.), Oil (q.s.), Salt (q.s.) | The leaves are cleaned, slightly fried in oil, added with other ingredients, ground and made as *chutney* | Once or twice with food | Anemia (1), Anorexia (1) |
|  | **Seeds of *Piper nigrum*** (2 g), **Seeds of *Cuminum cyminum*** (1 g), boiled rice (one cup), Clarified butter (q.s.) | The seeds are slightly roasted, powdered and added with rice and clarified butter | Once a day with lunch, for 3 – 5 days | Anemia (1), Anorexia (1) |
|  | **Tender fruits of *Trichosanthes cucumerina*** (100 g), Dehusked seeds of *Vigna radiata* (50 g), Bulbils of *Allium sativum* (q.s.), Bulbils of *Allium cepa* (q.s.), Leaves of *Murraya koenigii* (q.s.), Dried fruits of *Capsicum annuum* (q.s.), Oil (q.s.), Salt (q.s.) | The ingredients are made into gravy | Once with lunch, for 10 days | Oliguria (1) |
|  | **Rhizomes of *Beta vulgaris*** (50 g), Honey (q.s.) | The juice is consumed with honey | Once in the morning for a week | Anemia (1) |
|  | **Seeds of *Pennisetum glaucum*** (100 g), **Hand pounded rice (*Oryza sativa*)** (50 g), **Seeds of *Vigna mungo*** (25 g), **Seeds of *Trigonella foenum-graecum*** (25 g) | The ingredients are soaked in water, ground well and made as a batter. It is fermented overnight and used to prepare *dosa* | As breakfast | Anemia (1) |
|  | **Leaves of *Portulaca quadrifida*** (100 g), Dehusked seeds of *Vigna radiata* (50 g), Bulbils of *Allium sativum* (q.s.), Bulbils of *Allium cepa* (q.s.), Leaves of *Murraya koenigii* (q.s.), Dried fruits of *Capsicum annuum* (q.s.), Oil (q.s.), Salt (q.s.) | The leaves are cleaned and boiled with green gram. The other ingredients are chopped/powdered and fried in clarified butter. Both are mixed and smashed | Once a day, twice a week | Dysuria (2), Gastric ulcers (2), Hypertension (1), Anemia (1) |
|  | **Tender fruit juice of  *Cucumis sativus*** (100 mL), Salt (q.s.) | Consumed when needed | Once a day for 5 days | Hyperacidity (2) |
|  | **Flowers of *Musa paradisiaca*** (100 g), Dehusked seeds of *Vigna radiata* (50 g), Bulbils of *Allium sativum* (q.s.), Bulbils of *Allium cepa* (q.s.), Leaves of *Murraya koenigii* (q.s.), Dried fruits of *Capsicum annuum* (q.s.), Oil (q.s.), Salt (q.s.) | The flowers are cleaned and boiled with green gram. The other ingredients are chopped/powdered and fried in clarified butter. Both are mixed and smashed | Once a day with lunch for 30 days | Hemorrhoids (1) |
|  | Boiled rice (*Oryza sativa*) (200 g), **Grated endosperms of *Cocos nucifera*** (100 g), Dried fruits of *Capsicum annuum* (q.s.), Oil (q.s.), Salt (q.s.) | The ingredients are fried in oil and mixed with boiled rice | Once a day | Gastric ulcers (1), Mouth ulcers (1) |
|  | **Seeds of *Cuminum cyminum*** (1 – 2 g) Boiled rice of *Oryza sativa* | Boiled rice is fermented overnight with adequate water and the water is consumed with cumin seeds in the morning | Once in the morning for 15 days | Body heat (2), Hypertension (1), Gastric ulcers (2) |
|  | **Leaves of *Spinacia oleracea*** (100 g), Dehusked seeds of *Vigna radiata* (50 g), Bulbils of *Allium sativum* (q.s.), Seeds of *Cuminum cyminum* (q.s.), Seeds of *Piper nigrum* (q.s.), Dried fruits of *Capsicum annuum* (q.s.), Salt (q.s.) | The ingredients are made into gravy. | Twice a week | Hypertension (1) |
|  | **Leaves of *Cichorium intybus*** (200 g), Dehusked seeds of *Vigna radiata* (50 g), Bulbils of *Allium sativum* (q.s.), Bulbils of *Allium cepa* (q.s.), Leaves of *Murraya koenigii* (q.s.), Dried fruits of *Capsicum annuum* (q.s.), Clarified butter (q.s.), Salt (q.s.) | The leaves are cleaned, chopped and boiled with green gram. The other ingredients are chopped/powdered and fried in clarified butter. Both are mixed and smashed | Once a day with lunch, twice a week for three months | Impotence (1) |
|  | **Leaves of *Solanum americanum*** (50 g), Grated endosperm of *Cocos nucifera* (25 g), Dry fruits of *Capsicum annuum* (q.s.), Seeds of *Cuminum cyminum* (q.s.), Oil (q.s.), Salt (q.s.) | The leaves are cleaned, boiled and added with other ingredients and slightly fried. | Once a day, thrice a week for a month | Gastric ulcers (2), Mouth ulcers (2), Fever (1), Somatalgia (1), Fatigue (1), Convulsions (1), Headache (1), Constipation (1), Liver diseases (1), Jaundice (1), Mumps (1), Cough (1), Dermatological ailments (1), Hypertension (1) |
|  | **Leaves of *Alternanthera sessilis*** (50 g), Grated endosperm of *Cocos nucifera* (25 g), Dry fruits of *Capsicum annuum* (q.s.), Seeds of *Cuminum cyminum* (q.s.), Clarified butter (q.s.), Salt (q.s.) | The leaves are cleaned, boiled and added with other ingredients and slightly fried. | Once with lunch for 45 days | To increase memory and vision (1) |
|  | **Leaves of *Eclipta prostrata*** (250 g), Dehusked seeds of *Vigna radiata* (50 g), Bulbils of *Allium sativum* (q.s.), Bulbils of *Allium cepa* (q.s.), Leaves of *Murraya koenigii* (q.s.), Dried fruits of *Capsicum annuum* (q.s.), Clarified butter (q.s.), Salt (q.s.) | The ingredients are made into gravy. | Once with lunch for 45 days | To increase memory (1), Liver ailments (2), Anemia (2) |
|  | **Leaves of *Solanum trilobatum*** (250 g), Grated endosperm of *Cocos nucifera* (25 g), Dry fruits of *Capsicum annuum* (q.s.), Seeds of *Cuminum cyminum* (q.s.), Clarified butter (q.s.), Salt (q.s.) | The leaves are cleaned, boiled and added with other ingredients and slightly fried. | Once a day for 15 days | To increase memory (1) |
|  | **Flour of *Eleusine coracana*** (25 g), Milk of  *Bos taurus* (q.s.), Sugar from *Borassus flabellifer* (q.s.) | The flour is made into a porridge with milk and palm sugar | Once in the morning as needed | To strengthen memory (1) |
|  | **Roasted seeds of *Sesamum indicum*** (200 g), **Seeds of *Nigella sativa*** (25 g), Seeds of *Cuminum cyminum* (25 g), Sugar from *Borassus flabellifer* (200 g) | The ingredients slightly fried, pounded with palm sugar and made as small balls, approximately having 25 g | 1 – 2 balls, twice a day for three days | Amenorrhea (1) |
|  | **Fruit juice of *Citrullus lanatus*** (75 mL), Sugar from *Borassus flabellifer* (q.s.) | The ingredients are mixed and consumed | Once a day for five days | Hyperacidity (2), Dysuria (1) |
|  | **Fruits *Citrus medica***, Honey (in equal amounts) | The fruits are cut into pieces and pickled in honey | 10 – 20 g., with food | Hyperacidity (1) |
|  | **Fruits of *Psidium guajava*** | The fruits are consumed as raw | When available | Hyperacidity (1), Constipation (2), Diabetes (1) |
|  | **Fruits juice of *Solanum lycopersicum*** (50 mL), Honey (q.s.) | The ingredients are mixed and consumed | Once in the evening | Constipation (1), Heart disease (1), Diabetes (1), Anorexia (1), Rheumatalgia (1), To increase vision (1) |
|  | **Fruit juice of *Mangifera indica*** (50 mL), Milk (50 mL), Honey (q.s.) | The ingredients are mixed and consumed | Once in the morning after food for 30 days | Impotence (2), Heart diseases (4), Anorexia (3), To increase vision (1) |
|  | **Ripe fruits of *Carica papaya*** | The fruits are consumed | Once in the evening | Constipation (1) |
|  | **Pulp from the fruits of *Annona squamosa*** Jaggery from *Borassus flabellifer* (in equal quantities) | The ingredients are made into a jam | 1 – 2 g., once a day with milk | Coolant (1), Gastric ulcers (1) |
|  | **Fruit juice of *Citrus medica*** Jaggery from *Borassus flabellifer* (in equal quantities) | The ingredients are made into syrup | 1 – 2 g., before food | Heart disease (2), Gastric ulcers (2), Hypertension (2), Anorexia (2), Dermatological ailments (1) |
|  | **Fruit juice of *Citrus reticulata*** (100 g), Honey (100 mL) | The ingredients are made into syrup | 10 mL., twice a day | Heart diseases (1) |
|  | **Fruit pulp of *Ziziphus jujuba*** (50 g), Dried fruits of *Capsicum annuum* (q.s.), Salt (q.s.) | The ingredients are ground well as *chutney* | Once a day with food | Anorexia (3), Nausea (1), Diabetes (1) |
|  | **Dried rhizome of *Zingiber officinale*** (50 g), Seeds of *Coriandrum sativum* (50 g), Jaggery from *Borassus flabellifer* (q.s.) | Ginger and coriander seeds are powdered, about 3 g of the powder is made into tea and consumed with palm sugar | 1 – 2 times | Bloating (1) |
|  | **Flowers of *Musa paradisiaca*** (100 g), Dehusked and roasted seeds of *Vigna radiata* (30 g), Bulbils of *Allium cepa* (5 g), Fresh leaves of *Murraya koenigii* (5 g), Fresh leaves of *Coriandrum sativum* (5 g), Dried fruits of *Capsicum annuum* (q.s.), Salt (q.s.) | The tender flowers are cleaned and ground with other ingredients as *chutney*. | Once with lunch for 30 days | Hemorrhoids (1), Bleeding through rectum (1) |
|  | **Leaves of *Murraya koenigii*** (10 g), Seeds of *Cajanus cajan* (50 g), Fresh rhizome of *Zingiber officinale* (2 g), Seeds of *Cuminum cyminum* (1 g), Seeds of *Piper nigrum* (1 g), Seeds of *Trigonella foenum-graecum* (2 g), Resin of *Ferula assa-foetida* (q.s.), Salt (q.s.) | The ingredients are slightly roasted and powdered. | 5 g., with ghee and rice; Once in a lunch for a week | Indigestion (1), Bloating (1) |
|  | **Leaves of *Trianthema portulacastrum*** (50 g), Seeds of *Cajanus cajan* (25 g), Fresh rhizome of *Zingiber officinale* (q.s.), Seeds of *Cuminum cyminum* (q.s.), Seeds of *Piper nigrum* (q.s.), Seeds of *Trigonella foenum-graecum* (q.s.), Resin of *Ferula assa-foetida* (q.s.), Salt (q.s.), Clarified butter (q.s.) | The leaves are cleaned and boiled with pigeon pea. The other ingredients are crushed, fried with clarified butter, mixed with boiled leaves and smashed. | Once with lunch, twice a week for three months | Heart diseases (1) |
|  | **Fresh leaves of *Acalypha indica*** (25 g), Raw rice of *Oryza sativa* (100 g), Dehusked seeds of *Vigna radiata* (50 g), Salt (q.s.) | Rice and black gram seeds are made in to batter and mixed with the leaves and made as *dosa.* | 1 – 2 *dosas,* in the morning for a week | Bronchitis (1), Hemorrhoids (2), Intestinal worms (1) |
|  | **Leaves of *Melochia corchorifolia*** (50 g), Seeds of *Cajanus cajan* (25 g), Fresh rhizome of *Zingiber officinale* (q.s.), Seeds of *Cuminum cyminum* (q.s.), Seeds of *Piper nigrum* (q.s.), Seeds of *Trigonella foenum-graecum* (q.s.), Resin of *Ferula assa-foetida* (q.s.), Salt (q.s.), Clarified butter (q.s.) | The leaves are cleaned and boiled with pigeon pea. The other ingredients are crushed, fried with clarified butter, mixed with boiled leaves and smashed. | Once a day with lunch for a week | Pain during menopause (1) |
|  | **Tender leaves of *Cleome gynandra*** (20 g), Fresh rhizome of *Zingiber officinale* Roscoe (10 g), Seeds of *Cuminum cyminum* (q.s.), Seeds of *Piper nigrum* (q.s.), Salt (q.s.) | Cleaned leaves and ginger are slightly fried in clarified butter and ground well with other ingredients as *chutney* | Once in the morning for two days | Joint pain (1), Rhinitis (2), Fever (1), Headache (1) |
|  | **Fresh leaves of *Ipomoea aquatica*** (50 g), Dehusked seeds of *Cajanus cajan* (30 g), Bulbils of *Allium sativum* (q.s.), Bulbils of *Allium cepa* (q.s.), Leaves of *Murraya koenigii* (q.s.), Dried fruits of *Capsicum annuum* (q.s.), Clarified butter (q.s.), Salt (q.s.) | Cleaned leaves are boiled with pigeon pea; the other ingredients are crushed and fried with clarified butter, mixed with boiled leaves and smashed. | Once with lunch, twice a week for three months | Mouth ulcers (1), Gastric ulcers (2), Oligospermia (2) |
|  | **Fresh leaves of *Digera muricata*** (50 g), Dehusked seeds of *Cajanus cajan* (25 g), Bulbils of *Allium sativum* (q.s.), Bulbils of *Allium cepa* (q.s.), Leaves of *Murraya koenigii* (q.s.), Dried fruits of *Capsicum annuum* (q.s.), Oil (q.s.), Salt (q.s.) | Cleaned leaves are boiled with pigeon pea; the other ingredients are crushed and fried with oil, mixed with boiled leaves and smashed. | Once with lunch for 1 – 2 days | Diarrhea (1), Dysentery (1) |
|  | **Fresh, tender leaves of *Abutilon indicum*** (50 g), Dehusked seeds of *Cajanus cajan* (20 g), Bulbils of *Allium sativum* (q.s.), Bulbils of *Allium cepa* (q.s.), Leaves of *Murraya koenigii* (q.s.), Dried fruits of *Capsicum annuum* (q.s.), Oil (q.s.), Salt (q.s.) | Cleaned leaves are boiled with pigeon pea; the other ingredients are crushed and fried with oil, mixed with boiled leaves and smashed. | Once with lunch, twice a week | Hemorrhoids (3) |
|  | **Fresh leaves of *Boerhavia diffusa*** (30 g), Dehusked seeds of *Cajanus cajan* (30 g), Bulbils of *Allium sativum* (q.s.), Bulbils of *Allium cepa* (q.s.), Leaves of *Murraya koenigii* (q.s.), Dried fruits of *Capsicum annuum* (q.s.), Oil (q.s.), Salt (q.s.) | Cleaned leaves are boiled with pigeon pea; the other ingredients are crushed and fried with oil, mixed with boiled leaves and smashed. | Once a day for 15 days | Dysuria (3) |
|  | **Fresh leaves of *Marsilea quadrifolia*** (50 g), Dehusked seeds of *Cajanus cajan* (30 g), Bulbils of *Allium sativum* (q.s.), Bulbils of *Allium cepa* (q.s.), Leaves of *Murraya koenigii* (q.s.), Dried fruits of *Capsicum annuum* (q.s.), Oil (q.s.), Salt (q.s.) | Cleaned leaves are boiled with pigeon pea; the other ingredients are crushed and fried with oil, mixed with boiled leaves and smashed. | Once a day for 15 days | Polyuria (2), Ulcers in urinary tract (2) |
|  | **Fresh leaves of *Tribulus terrestris*** (50 g), Dehusked seeds of *Cajanus cajan* (30 g), Bulbils of *Allium sativum* (q.s.), Bulbils of *Allium cepa* (q.s.), Leaves of *Murraya koenigii* (q.s.), Dried fruits of *Capsicum annuum* (q.s.), Oil (q.s.), Salt (q.s.) | Cleaned leaves are boiled with pigeon pea; the other ingredients are crushed and fried with oil, mixed with boiled leaves and smashed. | Once a day for a month | Dysuria (2), Oligospermia (2), Burning sensation (1) |
|  | **Fresh leaves of *Cardiospermum halicacabum*** (50 g), Seeds of *Cajanus cajan* (20 g), Bulbils of *Allium cepa* (q.s), Fresh leaves of *Murraya koenigii*. (q.s), Fresh leaves of *Coriandrum sativum* (q.s), Dried rhizomes of *Curcuma longa* (q.s), Dried fruits of *Capsicum annuum* (q.s), Dried seeds of *Coriandrum sativum* (q.s), Oil of *Arachis hypogaea* (q.s), Fruits of *Solanum lycopersicum* (q.s), Salt (q.s) | The leaves are cleaned, chopped and made into gravy (*sambar*) | Once with lunch for 1 – 2 days | Somatalgia (2) |
|  | **Fresh flowers of *Sesbania grandiflora*** (100 g), Seeds of *Cajanus cajan* (20 g), Bulbils of *Allium cepa* (q.s), Fresh leaves of *Murraya koenigii* (q.s), Fresh leaves of *Coriandrum sativum* (q.s), Dried rhizomes of *Curcuma longa* (q.s), Dried fruits of *Capsicum annuum* (q.s), Dried seeds of *Coriandrum sativum* (q.s), Oil of *Arachis hypogaea* (q.s), Fruits of *Solanum lycopersicum* (q.s), Salt (q.s) | The ingredients are made into a gravy (*sambar*) | Once with lunch for 1 – 2 days | Burning sensation (1), Dysuria (1), Hypertension (1) |
|  | **Fresh flowers of *Senna auriculata*** (50 g), Dehusked seeds of *Vigna radiata* (25 g), Bulbils of *Allium sativum* (q.s.), Bulbils of *Allium cepa* (q.s.), Leaves of *Murraya koenigii* Seeds of *Piper nigrum* (q.s), Dried fruits of *Capsicum annuum* (q.s.), Clarified butter (q.s.), Salt (q.s.) | The flowers are cleaned and boiled with mung beans; the other ingredients are crushed and fried in clarified butter, mixed with the flowers and smashed. | Once a day with lunch, twice a week | Diabetes (2), Burning sensation (1) |
|  | **Flowers of *Cassia fistula*** (50 g), Dehusked seeds of *Vigna radiata* (25 g), Bulbils of *Allium sativum* (q.s.), Bulbils of *Allium cepa* (q.s.), Leaves of *Murraya koenigii* (q.s.), Seeds of *Piper nigrum* (q.s), Dried fruits of *Capsicum annuum* (q.s.), Clarified butter (q.s.), Salt (q.s.) | The flowers are cleaned and boiled with mung beans; the other ingredients are crushed and fried in clarified butter, mixed with the flowers and smashed. | Once a day with lunch, twice a week | Diabetes (1) |
|  | **Flowers of *Solanum trilobatum*** (10 g), Milk (q.s.), Honey (q.s.) | The leaves are boiled in honey and consumed | Once in the evening for 40 days | Impotence (2), Oilgospermia (1) |
|  | **Flowers of *Tamarindus indica*** (50 g), Dehusked seeds of *Vigna radiata* (25 g), Bulbils of *Allium sativum* (q.s.), Bulbils of *Allium cepa* L. (q.s.), Leaves of *Murraya koenigii* Spreng. (q.s.), Seeds of *Piper nigrum* (q.s), Dried fruits of *Capsicum annuum* (q.s.), Clarified butter (q.s.), Salt (q.s.) | The ingredients are made into a gravy | Twice a week, with lunch | Anorexia (1), Nausea (1) |
|  | **Tender fruits of *Coccinia grandis*** (50 g), Dehusked seeds of *Cajanus cajan* (20 g), Bulbils of *Allium cepa* (q.s), Fresh leaves of *Murraya koenigii*. (q.s), Fresh leaves of *Coriandrum sativum* (q.s), Dried rhizomes of *Curcuma longa* (q.s), Dried fruits of *Capsicum annuum* (q.s), Dried seeds of *Coriandrum sativum* (q.s), Oil of *Arachis hypogaea* (q.s), Fruits of *Solanum lycopersicum* (q.s), Salt (q.s) | The ingredients are made into gravy (*sambar*) | Twice – thrice a week | Bronchitis (1), Diabetes (2) |
|  | **Tender fruits of *Brassica oleracea* var. *gongylodes*** (50 g), Dehusked seeds of *Cajanus cajan* (20 g), Bulbils of *Allium cepa* (q.s), Fresh leaves of *Murraya koenigii* (q.s), Fresh leaves of *Coriandrum sativum* (q.s), Dried rhizomes of *Curcuma longa* (q.s), Dried fruits of *Capsicum annuum* (q.s), Dried seeds of *Coriandrum sativum* (q.s), Oil of *Arachis hypogaea* (q.s), Fruits of *Solanum lycopersicum* (q.s), Salt (q.s) | The ingredients are made into gravy (*sambar*) | Twice – thrice a week | Diabetes (2) |
|  | **Fruits of *Benincasa hispida*** (100 g), Dehusked seeds of *Cajanus cajan* (30 g), Bulbils of *Allium cepa* (q.s), Fresh leaves of *Murraya koenigii* Spreng. (q.s), Fresh leaves of *Coriandrum sativum* (q.s), Dried rhizomes of *Curcuma longa* (q.s), Dried fruits of *Capsicum annuum* (q.s), Dried seeds of *Coriandrum sativum* (q.s), Oil of *Arachis hypogaea* (q.s), Fruits of *Solanum lycopersicum* (q.s), Salt (q.s) | The ingredients are made into gravy (*sambar*) | Twice – thrice a week | Dysuria (1), To gain weight (1) |
|  | **Tender fruits of *Abelmoschus esculentus*** (50 g) | The fruits are cut, soaked in water overnight and the water is consumed in the morning | Once a day for 30 days | Diabetes (2), Body heat (1), oligospermia (1) |
|  | **Tender leaves of *Hibiscus cannabinus*** (50 g), unripe fruits of *Capsicum annuum* (q.s.), Salt (q.s.) | The leaves are fried in oil and ground with other ingredients as *chutney* | Consumed with food, twice a day | Anorexia (1) |
|  | **Tender leaves of *Oxalis corniculata*** (50 g), unripe fruits of *Capsicum annuum* (q.s.), Salt (q.s.) | The ingredients are ground and made into *chutney* | Consumed with food, twice a day | Hypertension (2), Insomnia (1) |
|  | **Tender, unripe fruits of *Carica papaya*** (100 g), Dehusked seeds of *Vigna radiata* (25 g), Bulbils of *Allium sativum* (q.s.), Bulbils of *Allium cepa* (q.s.), Leaves of *Murraya koenigii* (q.s.), Seeds of *Piper nigrum* (q.s), Dried fruits of *Capsicum annuum* (q.s.), Clarified butter (q.s.), Salt (q.s.) | The ingredients are made into gravy | Consumed once a day, twice a week | Lactogogue (1), joint pain (1) |
|  | **Leaves of *Moringa oleifera*** (100 g), Flour of ***Eleusine coracana*** (100 g), Bulbils of *Allium cepa* (50 g), Dried fruits of *Capsicum annuum* (q.s.), Salt (q.s.) | The ingredients are added with little water and made as *roti* (flatbread) | Twice a week | Anemia (2) |
|  | **Milk of *Capra aegagrus hircus*** | The milk is consumed as raw | Once a day | Oligospermia (1) |
|  | **Intestine of *Capra aegagrus hircus*** | Cleaned, cooked and consumed | Once a week | Gastric ulcers (1) |
|  | **Testes  *Capra aegagrus hircus*** (50 g), Ghee (q.s.), Jaggery from *Borassus flabellifer* (50 g) | The testes are chopped, boiled added with other ingredients and made into a jam | Once in night times, with milk for 15 days | Importance (1) |
|  | **Hoofs of *Capra aegagrus hircus*** | Cleaned, cooked and consumed | Once a week | Bone fractures (2) |
|  | **Milk of  *Bos taurus*** (100 mL), Honey (q.s.) | With milk, honey is added and consumed | Once in the morning | To increase memory (1) |
|  | **Butter milk of *Bos taurus*** | The buttermilk is consumed | As required | Burning sensation (2), Oliguria (1), Indigestion (1) |
|  | **Eggs of**  ***Gallus gallus domesticus*** (2 Nos.), **Leaves of *Moringa oleifera*** (50 g), Bulbils of *Allium cepa* (25 g), Dry fruits of *Capsicum annuum* (q.s.), Oil of *Arachis hypogaea* (q.s.), Salt (q.s.) | The leaves are cooked and fried with other ingredients | Consumed with food, once a day | Anemia (2) |
|  | **Eggs of**  ***Gallus gallus domesticus*** | Cooked and consumed | Once a day | Bronchitis for children (1) |
|  | ***Rastrelliger kanagurta*** | The fishes are cleaned and made as gravy. | With food, twice a week | Bronchitis (3) |
|  | ***Gerres oyena*** | The fishes are cleaned and made as gravy. | With food once a week | Somatalgia (1), Bronchitis (1), Lactogogue (1) |
|  | ***Uroteuthis duvauceli*** | Cleaned and made as gravy. | With food thrice a week | Anemia (2), Rheumatalgia (1), Bronchitis (1) |
|  | ***Portunus sanguinolentus*** | The crabs are cleaned and made as gravy. | Once a week | Bronchitis (3), Fever (2) |
|  | ***Gibelion catla*** | The fishes are cleaned and fried in oil. | Once a week | Fatigue (2), Somatalgia (1), Impotence (1), Lactogogue (2), To increase vision (1) |
|  | ***Lebeo rohita*** | The fishes are cleaned and fried in oil. | Once a week | Somatalgia (1), Heart disease (1), Eye disease (1), Obesity (1) |
|  | ***Parastromateus niger*** | The fishes are cleaned and made into gravy | Twice a week | Wheezing (3), to increase memory (1) |
|  | ***Eleutheronema tetradactylum*** | The fishes are cleaned and made into gravy | Once a week | Impotence (1), Heart diseases (1), Hypertension (1), Urolithiasis (2) |
|  | ***Mystus* sp.** | The fishes are cleaned and made into gravy | Twice a week | Bronchitis (1) |
|  | ***Lates calcarifer*** | The fishes are cleaned and made into gravy | Once a week | Rheumatalgia (3), Impotence (1) |
|  | ***Nemipterus japonicus*** | The fishes are cleaned and made into gravy | Twice a week | Chest pain (1) |
|  | ***Sardinella longiceps*** | The fishes are cleaned and made into gravy | Twice a week | Anabolic (4), Fatigue (1) |
|  | ***Katelysia opima*** | The fleshy parts are made into gravy | Once a week | Bronchitis (1), Impotence (2) |
|  | ***Oreochromis mossambicus*** | The fleshy parts are made into gravy | Once a week | Bronchitis (1), Joint pain (1), Fatigue (1), lactogogue (1), Chest pain (1) |
|  | ***Parupeneus indicus*** | The fleshy parts are made into gravy | Once a week | Coolant (1) |
|  | ***Parathelphusa hydrodromus*** | The crabs are cleaned and made in to soup | Once a day | Fever (3) |
|  | ***Himantura uarnak*** | The fishes are cleaned and made as a gravy | Once or twice a week | Somatalgia (1), wheezing (3) |
|  | ***Stolephorus* spp.** | The fishes are made into curry. | Once a week | Oligospermia (2) |
|  | ***Upeneus sulphureus*** | The fishes are made into curry. | Once a week | Joint pain (2) |
|  | ***Lactarius lactarius*** | The fishes are made into curry. | Whenever needed | Chest pain (1) |
|  | ***Euthynnus affinis*** | The fishes are cleaned and made as a gravy | Once or twice a week | Fever (1), Cough (2) |
|  | ***Stolephorus indicus*** | The fishes are cut and cleaned into the water. And made into fish curry. | Twice a week | Cough (1), Wheezing (2), Lactogogue (2) |
|  | ***Rachycentron canadus*** | The fishes are cleaned and made into curry | Twice a week | Good for pregnant women (1), Lactogogue (1) |
|  | ***Chanos chanos*** | The fishes are cleaned and made into gravy | Twice a week | Lactogogue (1), Fatigue (1), Impotence (1) |
|  | ***Scoliodon laticaudus*** | Cleaned parts are fried and consumed | Twice a week | Lactogogue (7) |
|  | ***Caranx melampygus*** | The fishes are cleaned and made into gravy | Once a week | Anabolic (1), Cough (1), Chest pain (1), Wheezing (1), Coolant (1) |
|  | ***Monodactylus argenteus*** | The fishes are cleaned and made into gravy | Once a week | Cough (1) |
|  | ***Thryssa malabarica*** | The fishes are cleaned and made into gravy | Once a week | Arthritis (1) |
|  | ***Scomberomorus guttatus*** | Cleaned, fried and consumed | Once a week with food | Obesity (1), Fatigue (1), Chest pain (1), Rheumatalgia (1) |
|  | ***Trichiurus lepturus* Linnaeus**  ***Eupleurogrammus muticus* (Gray)** | The fishes are cleaned and made into gravy | Once a week | Giddiness (1), Bronchitis (2), Joint pain (1) |
|  | ***Channa* spp.** | The fishes are cut into small pieces and cleaned into the water. The fishes are made into gravy. | The gravy is consumed with rice, once a day for 15 days | Anabolic (1), Oligospermia (1), Coolant (1) |
|  | ***Anguilla bengalensis bengalensis*** | Made as gravy and consumed | Twice a week | Fatigue (1), Joint pain (1) |
|  | ***Fenneropenaeus indicus*** | Cleaned, made as gravy and consumed | Once a week | To improve vision (1), Anabolic (3), Lactogogue (1) |
|  | ***Pampus argenteus*** | Cleaned, made as gravy and consumed | Once a week | Cough (2), Lactogogue (1) |
|  | ***Sphyraena jello*** | Cleaned, made as gravy and consumed | Once a week | Lactogogue (1), Anabolic (2), Coolant (1) |

The taxa given bold are mentioned as the key taxa for the reported medicinal activity of the food by the informants; Values given with in the parentheses indicate the number of UR for the respective illness

**Table S2**: Cultural Food Significance Index of the plant and animal taxa cited by the non-institutionally trained *siddha* practitioners of Tiruvallur district of Tamil Nadu for preparing medicinal foods

| **Name of the taxa** | **AI** | **QI** | **UFI** | **PUI** | **MFFI** | **TSAI** | **FMRI** | **CFSI** |
| --- | --- | --- | --- | --- | --- | --- | --- | --- |
| ***Plants*** |  |  |  |  |  |  |  |  |
| *Abelmoschus esculentus* | 3 | 4 | 4 | 1 | 1.5 | 5.5 | 4 | 15.84 |
| *Abutilon indicum* | 2.5 | 3 | 3 | 1.5 | 1.5 | 6.5 | 5 | 16.45 |
| *Acalypha indica* | 2.5 | 4 | 3 | 1.5 | 1.5 | 5.5 | 4 | 14.85 |
| *Allium cepa* | 3 | 7 | 5 | 1.5 | 0.65 | 7.5 | 5 | 38.39 |
| *Allium sativum* | 3 | 5 | 5 | 1.5 | 0.65 | 6.5 | 4 | 19.01 |
| *Aloe vera* | 1.5 | 8 | 3 | 1.5 | 1.31 | 4 | 4 | 11.31 |
| *Alternanthera sessilis* | 3 | 3 | 4 | 1.5 | 0.87 | 6.5 | 3 | 9.16 |
| *Amaranthus viridis* | 2.5 | 4 | 3 | 1.5 | 1.5 | 7.5 | 4 | 20.25 |
| *Amorphophallus paeoniifolius* | 3.5 | 4 | 4 | 1.5 | 1.37 | 6.5 | 5 | 37.40 |
| *Anacardium occidentale* | 3 | 3 | 4 | 1 | 1 | 7.5 | 4 | 10.80 |
| *Annona squamosa* | 3.5 | 2 | 3 | 1 | 1 | 7.5 | 3 | 4.72 |
| *Arachis hypogaea* | 3 | 3 | 4 | 1 | 1 | 7.5 | 4 | 10.80 |
| *Benincasa hispida* | 2 | 2 | 3 | 1 | 1.5 | 6.5 | 3 | 3.51 |
| *Beta vulgaris* | 3 | 4 | 5 | 1 | 0.87 | 6.5 | 4 | 13.57 |
| *Boerhavia diffusa* | 1.5 | 3 | 2 | 1.5 | 1.5 | 5.5 | 5 | 5.56 |
| *Borassus flabellifer* | 3.5 | 4 | 3 | 1 | 1 | 7.5 | 5 | 15.75 |
| *Brassica oleracea var. gongylodes* | 2 | 2 | 4 | 1.5 | 1.5 | 7.5 | 5 | 13.50 |
| *Cardiospermum halicacabum* | 2.5 | 5 | 3 | 1.5 | 1 | 9 | 5 | 25.31 |
| *Carica papaya* | 3 | 3 | 4 | 1 | 1.1 | 7.5 | 3 | 8.91 |
| *Centella asiatica* | 2 | 3 | 3 | 1.5 | 1.5 | 9 | 4 | 14.58 |
| *Cicer arietinum* | 2.5 | 4 | 5 | 1 | 1 | 6.5 | 4 | 13.00 |
| *Cissus quadrangularis* | 3 | 6 | 3 | 1 | 1.5 | 10 | 4 | 32.40 |
| *Citrullus lanatus* | 2 | 3 | 3 | 1 | 0.75 | 6.5 | 4 | 3.51 |
| *Citrus limon* | 3 | 11 | 4 | 1 | 1 | 7.5 | 4 | 39.60 |
| *Citrus medica* | 2.5 | 10 | 3 | 1 | 1 | 6.5 | 5 | 24.37 |
| *Cleome gynandra* | 2.5 | 5 | 2 | 1.5 | 1.5 | 9 | 4 | 20.25 |
| *Coccinia grandis* | 2.5 | 14 | 3 | 1 | 1.16 | 7.5 | 5 | 45.67 |
| *Cocculus hirsutus* | 2.5 | 2 | 2 | 1.5 | 1 | 4 | 3 | 1.80 |
| *Cocos nucifera* | 3 | 4 | 5 | 1 | 1 | 6.5 | 3 | 11.70 |
| *Cucumis sativus* | 2 | 2 | 3 | 1 | 1 | 6.5 | 3 | 2.34 |
| *Cuminum cyminum* | 3 | 8 | 5 | 1 | 1.33 | 10 | 4 | 63.84 |
| *Daucus carota* | 3 | 4 | 4 | 1.5 | 0.5 | 6.5 | 4 | 9.36 |
| *Digera muricata* | 1.5 | 2 | 3 | 1.5 | 1.5 | 5.5 | 5 | 5.56 |
| *Echinochloa frumentacea* | 2 | 3 | 3 | 1 | 1 | 5.5 | 4 | 3.96 |
| *Eclipta prostrata* | 2.5 | 6 | 3 | 1.5 | 1.5 | 7.5 | 4 | 30.37 |
| *Eleusine coracana* | 3 | 7 | 4 | 1 | 1 | 7.5 | 4 | 25.20 |
| *Ficus racemosa* | 2.5 | 8 | 3 | 1 | 0.93 | 7.5 | 4 | 16.74 |
| *Gossypium* spp*.* | 1.5 | 2 | 2 | 1 | 1 | 4 | 3 | 0.72 |
| *Hedyotis puberula* | 2 | 3 | 2 | 1.5 | 1.5 | 6.5 | 5 | 8.77 |
| *Hibiscus rosa-sinensis* | 2.5 | 3 | 2 | 1 | 1 | 7.5 | 4 | 4.50 |
| *Hybanthus enneaspermus* | 2 | 2 | 2 | 1 | 1.5 | 6.5 | 3 | 2.34 |
| *Ipomoea aquatica* | 2 | 5 | 3 | 1.5 | 1.5 | 6.5 | 5 | 21.93 |
| *Lagenaria siceraria* | 3 | 6 | 4 | 1 | 1.37 | 7.5 | 4 | 29.59 |
| *Leucas aspera* | 2 | 3 | 2 | 1 | 0.75 | 5.5 | 4 | 1.98 |
| *Limonia acidissima* | 2.5 | 5 | 2 | 1 | 1.5 | 7.5 | 5 | 14.06 |
| *Mangifera indica* | 2.5 | 10 | 3 | 1 | 0.75 | 7.5 | 5 | 21.09 |
| *Marsilea quadrifolia* | 2 | 4 | 2 | 1.5 | 1.5 | 5.5 | 5 | 9.90 |
| *Mentha arvensis* | 3 | 2 | 4 | 1.5 | 1.5 | 9 | 3 | 14.58 |
| *Momordica charantia* | 3 | 3 | 4 | 1 | 1 | 5.5 | 4 | 7.92 |
| *Moringa oleifera* | 3 | 18 | 4 | 1.5 | 0.82 | 9 | 4 | 95.64 |
| *Mukia maderaspatana* | 2.5 | 5 | 2 | 1.5 | 1.5 | 10 | 5 | 28.12 |
| *Murraya koenigii* | 3 | 8 | 5 | 1.5 | 1.5 | 9 | 4 | 97.20 |
| *Musa paradisiaca* | 3 | 11 | 3 | 1 | 1.5 | 7.5 | 5 | 55.68 |
| *Nigella sativa* | 2 | 2 | 2 | 1 | 1 | 6.5 | 3 | 1.56 |
| *Oryza sativa* | 3 | 5 | 5 | 1 | 1.1 | 7.5 | 4 | 24.75 |
| *Oxalis corniculata* | 2 | 3 | 3 | 1.5 | 1.5 | 7.5 | 4 | 12.15 |
| *Panicum sumatrense* | 3 | 3 | 4 | 1 | 1 | 6.5 | 4 | 9.36 |
| *Paspalum scrobiculatum* | 3 | 3 | 4 | 1 | 1 | 5.5 | 4 | 7.92 |
| *Pennisetum glaucum* | 3 | 4 | 4 | 1 | 1.12 | 6.5 | 4 | 13.97 |
| *Phoenix dactylifera* | 3 | 3 | 3 | 1 | 1 | 9 | 3 | 7.29 |
| *Phyllanthus emblica* | 3 | 14 | 3 | 1 | 1 | 9 | 4 | 45.36 |
| *Piper nigrum* | 3 | 3 | 5 | 1 | 0.91 | 6.5 | 3 | 7.98 |
| *Plectranthus amboinicus* | 2.5 | 2 | 3 | 1.5 | 0.5 | 4 | 5 | 2.25 |
| *Portulaca quadrifida* | 1 | 6 | 3 | 1 | 1.5 | 9 | 4 | 9.72 |
| *Prunus dulcis* | 3 | 3 | 3 | 1 | 1 | 6.5 | 4 | 7.02 |
| *Psidium guajava* | 3 | 4 | 3 | 1 | 0.75 | 6.5 | 5 | 8.77 |
| *Punica granatum* | 3 | 10 | 3 | 1 | 1.11 | 10 | 4 | 39.96 |
| *Senna auriculata* | 2.5 | 4 | 3 | 0.75 | 1.62 | 9 | 3 | 9.84 |
| *Sesamum indicum* | 3 | 3 | 3 | 1 | 0.75 | 6.5 | 3 | 3.94 |
| *Sesbania grandiflora* | 2.5 | 8 | 3 | 1.5 | 1 | 5.5 | 4 | 19.8 |
| *Setaria italica* | 3 | 3 | 3 | 1 | 1 | 5.5 | 4 | 5.94 |
| *Solanum americanum* | 3 | 23 | 4 | 1 | 1.11 | 9 | 4 | 110.28 |
| *Solanum lycopersicum* | 3 | 6 | 5 | 1 | 1.5 | 7.5 | 4 | 40.5 |
| *Solanum torvum* | 3 | 7 | 3 | 1 | 1.21 | 4 | 4 | 12.19 |
| *Solanum trilobatum* | 2.5 | 7 | 3 | 1.5 | 1 | 5.5 | 4 | 17.32 |
| *Sorghum bicolor* | 3.5 | 3 | 3 | 1 | 1 | 7.5 | 4 | 9.45 |
| *Syzygium cumini* | 2.5 | 2 | 3 | 1 | 0.5 | 5.5 | 5 | 2.06 |
| *Tamarindus indica* | 3 | 2 | 5 | 1.5 | 1.5 | 6.5 | 5 | 21.93 |
| *Trachyspermum ammi* | 3 | 2 | 4 | 1 | 0.75 | 4 | 3 | 2.16 |
| *Tribulus terrestris* | 3 | 5 | 2 | 1.5 | 1.5 | 7.5 | 4 | 20.25 |
| *Trigonella foenum-graecum* | 3 | 4 | 5 | 1 | 1.37 | 5.5 | 4 | 18.08 |
| *Vigna mungo* | 2 | 10 | 5 | 1 | 1.14 | 7.5 | 4 | 34.2 |
| *Vitis vinifera* | 3 | 9 | 3 | 1 | 1.38 | 9 | 4 | 40.24 |
| *Zea mays* | 3 | 3 | 3 | 1 | 1 | 7.5 | 4 | 8.1 |
| *Zingiber officinale* | 3 | 2 | 5 | 1.5 | 0.87 | 5.5 | 3 | 6.45 |
| *Ziziphus jujuba* | 1.5 | 5 | 3 | 1 | 1.5 | 7.5 | 4 | 10.12 |
| ***Animals*** |  |  |  |  |  |  |  |  |
| *Portunus sanguinolentus* | 3 | 5 | 4 | - | 1.5 | 7.5 | 5 | 33.75 |
| *Anguilla bengalensis bengalensis* | 1.5 | 2 | 2 | - | 1 | 7.3 | 3 | 1.31 |
| *Bos taurus* | 3 | 5 | 5 | - | 1 | 6.5 | 4 | 19.50 |
| *Capra aegagrus hircus* | 2 | 5 | 4 | - | 1 | 7.5 | 4 | 12.00 |
| *Caranx melampygus* | 1.5 | 5 | 2 | - | 1.5 | 7.5 | 4 | 6.75 |
| *Channa* spp*.* | 2 | 3 | 3 | - | 1.5 | 9 | 3 | 7.29 |
| *Chanos chanos* | 2.5 | 3 | 3 | - | 1.5 | 7.5 | 3 | 7.59 |
| *Eleutheronema tetradactylum* | 1.5 | 5 | 2 | - | 1.5 | 10 | 4 | 9.00 |
| *Euthynnus affinis* | 2 | 3 | 3 | - | 1.5 | 6.5 | 4 | 7.02 |
| *Fenneropenaeus indicus* | 3 | 5 | 4 | - | 1.5 | 7.5 | 4 | 27.00 |
| *Gallus gallus domesticus* | 3 | 3 | 4 | - | 1 | 6.5 | 4 | 9.36 |
| *Gerres oyena* | 1.5 | 3 | 2 | - | 1.5 | 7.5 | 3 | 3.03 |
| *Gibelion catla* | 2 | 7 | 3 | - | 1 | 7.5 | 4 | 12.60 |
| *Himantura uarnak* | 2 | 4 | 3 | - | 1.5 | 7.5 | 5 | 13.50 |
| *Katelysia opima* | 1.5 | 3 | 2 | - | 1.5 | 7.5 | 4 | 4.05 |
| *Lates calcarifer* | 1.5 | 4 | 2 | - | 1.5 | 7.5 | 5 | 6.75 |
| *Lebeo rohita* | 2 | 4 | 4 | - | 1 | 7.5 | 3 | 7.20 |
| *Oreochromis mossambicus* | 1.5 | 5 | 2 | - | 1.5 | 9 | 3 | 6.07 |
| *Pampus argenteus* | 2 | 3 | 3 | - | 1.5 | 7.5 | 4 | 8.10 |
| *Parastromateus niger* | 2 | 4 | 3 | - | 1.5 | 7.5 | 5 | 13.50 |
| *Parathelphusa hydrodromus* | 2 | 3 | 3 | - | 0.75 | 7.5 | 5 | 5.06 |
| *Rachycentron canadus* | 1.5 | 2 | 2 | - | 1 | 5.5 | 5 | 1.65 |
| *Rastrelliger kanagurta* | 3 | 3 | 3 | - | 1.5 | 9 | 5 | 18.22 |
| *Sardinella longiceps* | 3 | 5 | 4 | - | 1.5 | 6.5 | 5 | 29.25 |
| *Scoliodon laticaudus* | 2 | 7 | 3 | - | 1 | 7.5 | 5 | 15.75 |
| *Scomberomorus guttatus* | 2 | 4 | 3 | - | 1 | 6.5 | 3 | 4.68 |
| *Sphyraena jello* | 2.5 | 4 | 3 | - | 1.5 | 9 | 4 | 16.20 |
| *Stolephorus indicus* | 2 | 5 | 4 | - | 1.5 | 7.5 | 5 | 22.50 |
| *Trichiurus lepturus* &  *Eupleurogrammus muticus* | 1.5 | 4 | 2 | - | 1.5 | 7.5 | 3 | 4.05 |
| *Upeneus sulphureus* | 1.5 | 4 | 2 | - | 1 | 5.5 | 5 | 3.30 |
| *Uroteuthis duvauceli* | 2 | 2 | 3 | - | 1.5 | 6.5 | 4 | 4.68 |

Taxa having a minimum two UR was taken for the analysis; **AI**: Availability Index; **QI**: Quotation Index; **UFI**: Utilization Frequency Index; **PUI**: Parts Used Index; **MFFI**: Multi-Functional Food Use Index; **TASI**: Taste Score Appreciation Index; **FMRI**: Food-Medicinal Role Index; **CFSI**: Cultural Food Significance Index
